# A review on the pharmacology, pharmacokinetics and toxicity of sophocarpine

**DOI:** 10.3389/fphar.2024.1353234

**Published:** 2024-04-29

**Authors:** Shichao Wei, Junshen Xiao, Feng Ju, Jin Liu, Zhaoyang Hu

**Affiliations:** ^1^ Department of Anesthesiology, West China Hospital, Sichuan University, Chengdu, Sichuan, China; ^2^ Laboratory of Anesthesia and Critical Care Medicine, National-Local Joint Engineering Research Centre of Translational Medicine of Anesthesiology, West China Hospital, Sichuan University, Chengdu, Sichuan, China

**Keywords:** sophocarpine, pharmacology, pharmacokinetics, toxicity, review

## Abstract

Sophocarpine is a natural compound that belongs to the quinolizidine alkaloid family, and has a long history of use and widespread distribution in traditional Chinese herbal medicines such as *Sophora alopecuroides* L., *Sophora flavescens* Ait., and *Sophora subprostrata*. This article aims to summarize the pharmacology, pharmacokinetics, and toxicity of sophocarpine, evaluate its potential pharmacological effects in various diseases, and propose the necessity for further research and evaluation to promote its clinical application. A large number of studies have shown that it has anti-inflammatory, analgesic, antiviral, antiparasitic, anticancer, endocrine regulatory, and organ-protective effects as it modulates various signaling pathways, such as the NF-κB, MAPK, PI3K/AKT, and AMPK pathways. The distribution of sophocarpine in the body conforms to a two-compartment model, and sophocarpine can be detected in various tissues with a relatively short half-life. Although the pharmacological effects of sophocarpine have been confirmed, toxicity and safety assessments and reports on molecular mechanisms of its pharmacological actions have been limited. Given its significant pharmacological effects and potential clinical value, further research and evaluation are needed to promote the clinical application of sophocarpine.

## Introduction

**FIGURE 1:**
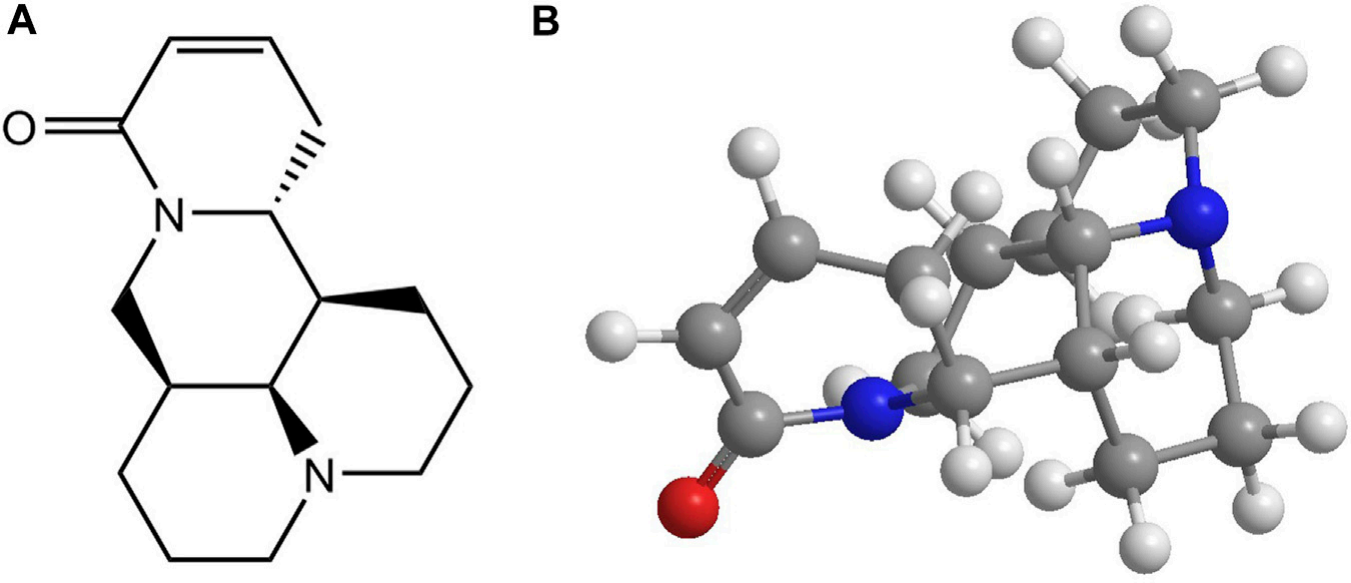
The molecular structure **(A)** and chemical 3D structure **(B)** of sophocarpine.

Natural products have played vital roles in pharmacotherapy. They can be obtained from various species ranging from mosses to flowering plants, animals, or microorganisms and have offered unique chemical templates or novel scaffolds for drug discovery (Atanasov et al., 2021). Many natural products have active pharmacological properties and possess therapeutic value (Guo et al., 2022). Historically, these medicinal plants have long been used for treating various human diseases in traditional Chinese medicine. Among such products, the alkaloids found in the bark, root, leaf, seed, flower, or other parts of the plant are among the largest and most important groups of naturally occurring organic molecules, and constitute the active ingredients of the crude medical source (Omar et al., 2021). Over 27,000 different alkaloids are listed in the Dictionary of Natural Products (DNP), and more than 70% of these are from plants. The genus *Sophora* exhibits a wide distribution across North America and Eurasia, encompassing several plant species that have long been employed in traditional herbal medicine, such as *Sophora alopecuroides* L. and *Sophora flavescens* Ait. The primary alkaloidal constituents of *Sophora* are presented in Table 1. Quinolizidine alkaloids, as the major active ingredients in the genus *Sophora*, are divided into different classes of alkaloids, including matrine-, lupinine-, lupanine-, macrocyclic bisquinolizidine-, biphenylquinolizidine lactone-, cytosine-, sparteine-, tetrahydrocytisine-, and anagyrine-type alkaloids. Among these compounds, matrine is the most common type, accounting for 13.6% of the total quinolizidine alkaloids (Cely-Veloza et al., 2023). Sophocarpine (C_15_H_22_N_2_O, CAS No. 145572-44-7) (Figure 1), also known as 13,14-didehydromatridin-15-one, is a tetracyclic matrine-type quinolizidine alkaloid found in *Sophora flavescens* Ait.*, Sophora alopecuroides* L.*, Sophora viciifolia*.*, Sophora tonkinensis* Gagnep., and *Daphniphyllum oldhamii* (Hemsl.) K. Rosenthal. These medicinal plants have been used in healthcare for centuries, especially in East Asia.

**TABLE 1:** The primary alkaloidal constituents of the genus *Sophora*.

Name	PubChem CID	Molecular formula	Biological and pharmacological effects	References
Sophocarpine	115269	C_15_H_22_N_2_O	Anti-inflammatory, anti-tumor, anti-virus, anti-parasitic, analgesia, anti-diabetes, multi-organ protection	Li et al. (2020)
Oxysophocarpine	24721085	C_15_H_22_N_2_O_2_	Anti-inflammatory, anti-tumor, anti-virus, neuroprotective, analgesia	Jiang et al. (2022)
Sophoridine	165549	C_15_H_24_N_2_O	Anti-inflammatory, anti-tumor, anti-virus, anti-bacterial activity, multi-organ protection	Wang et al. (2022a)
Oxysophoridine	71773433	C_15_H_24_N_2_O_2_	Anti-inflammatory, anti-tumor, anti-virus, multi-organ protection	Wang et al. (2013)
Matrine	91466	C_15_H_24_N_2_O	Anti-inflammatory, anti-tumor, anti-virus, anti-parasitic, multi-organ protection	Zhang et al. (2020)
Oxymatrine	114850	C_15_H_24_N_2_O_2_	Anti-inflammatory, anti-tumor, anti-virus, antidiabetic, multi-organ protection	Lan et al. (2020)
Aloperine	162147	C_15_H_24_N_2_	Anti-inflammatory, anti-tumor, anti-virus, anti-bacterial activity, multi-organ protection	Zhou et al. (2020)
Sophoramine	169014	C_15_H_20_N_2_O	Anti-inflammatory, anti-tumor, anti-arrhythmia	Bian and Toda (1988)
N-methylcytisine	670971	C_12_H_16_N_2_O	Anti-inflammatory, anti-virus	Jiao et al. (2018)
Cytisine	10235	C_11_H_14_N_2_O	Analgesia, reduce addiction to tobacco and alcohol, anti-tumor, anti-diabetes, anti-depression, multi-organ protection	Wang et al. (2024)

The beneficial effect of sophocarpine has been widely explored. Many modern pharmacological studies have revealed its multiple bioactive effects, such as anticancer (Luo et al., 2019), anti-inflammatory (Gao et al., 2012), antiviral (Gao et al., 2013; Sang et al., 2017), analgesic (Wang et al., 2021b), and organ protection (Li et al., 2014; Hou et al., 2020) or addressing vessel dysfunction (Fang et al., 2020) effects. Moreover, it also has an antidiabetic effect on type 1 diabetes (Su et al., 2021). Furthermore, sophocarpine exhibits great benefits in agriculture. Sophocarpine may be used as a natural control agent for the invasive and dangerous red imported fire ants (*Solenopsis invicta*) (Tian and Zhang, 2023). Meanwhile, sophocarpine also has aphicidal effects on pea aphids (*Acyrthosiphon pisum*) (Ma et al., 2020). In addition, sophocarpine is an ideal starting material for drug synthesis and structural modification due to its unique chemical structure with an α,β-unsaturated carbonyl group, which may provide a flexible reactive site for nucleophiles (Hu et al., 2010).

As a main bioactive component of quinolizidine alkaloids, sophocarpine has been widely studied in recent years. However, the pharmacological activities and underlying mechanism of sophocarpine have not been reviewed systematically. To further understand its progress, this review aims to provide up-to-date knowledge on the pharmacology, pharmacokinetics and toxicity of sophocarpine, hopefully providing valuable insights into its therapeutic potential in clinical settings. We believe this review will be helpful in understanding the key characteristics of sophocarpine and may offer constructive suggestions for further studies.

## Pharmacological activities

### Sophocarpine and inflammation

*Sophora alopecuroides* L. and its active components have a long history as a traditional folk medicine in China for the treatment of inflammation. Rheumatoid arthritis is a chronic systemic inflammatory autoimmune disease that can affect the joints (Sparks, 2019). Zhu’s group further confirmed that sophocarpine may be a potential drug for treating rheumatoid arthritis, demonstrating its ability to suppress the expression of proinflammatory cytokines and downregulate the MAPK and NF-κB signaling pathways (Zhu and Zhu, 2017). Similarly, Wu et al. have reported that sophocarpine can also attenuate inflammatory factors such as IL-6 and TNF-α in both *in vivo* and *in vitro* models of osteoarthritis-related inflammatory cartilage degeneration, with mechanisms involving the PI3K/AKT and NF-κB signaling pathways (Wu et al., 2019). Implant loosening can occur after long-term total joint arthroplasty. Using both *in vivo* and *in vitro* models, Zhou et al. reported that sophocarpine could prevent implant loosening by suppressing osteoclastogenesis and bone resorption. The molecular mechanism may involve the NF-κB signaling pathway. Therefore, sophocarpine may be used as a potential reagent for treating prosthesis loosening (Zhou et al., 2018). It is evident that targeting the NF-κB signaling pathway, along with upstream pathways such as PI3K/AKT and MAPK signaling pathways represents crucial therapeutic strategies for managing osteoarthritis using sophocarpine. However, it should be noted that other pathways such as the HIF2α (Saito et al., 2010) and Wnt/β-catenin (Monteagudo et al., 2022) pathways, have been demonstrated to significantly influence the pathogenesis of osteoarthritis. It remains uncertain whether these pathways directly contribute to the therapeutic effects of sophocarpine.

Lipopolysaccharide (LPS) is a component of the gram-negative bacterial outer membrane and may cause inflammation. The effect of sophocarpine on LPS-induced inflammatory responses in RAW 264.7 cells was investigated. The results indicated that sophocarpine has anti-inflammatory effects *in vitro*, which may be associated with the MAPK and NF-kB signaling pathways (Gao et al., 2012). Similarly, He et al. showed that sophocarpine reduced the levels of IL-6 and TNF-α in RAW 264.7 cells exposed to LPS and decreased the inflammatory response in zebrafish exposed to CuSO_4_, indicating that sophocarpine was effective in ameliorating inflammation both *in vitro* and *in vivo* (He et al., 2019). Ulcerative colitis is a chronic inflammatory bowel disease (Gajendran et al., 2019). Sophocarpine significantly ameliorated dextran sulfate sodium salt (DSS)- induced colitis by regulating pro- and anti-inflammatory cytokine production (Wang et al., 2012) and TLR4/MAPK and K2/STAT3 signaling pathway activation (Zhang et al., 2015). Similarly, Jiang et al. conducted a screening of five compounds (sophocarpine, sophoridine, cytisine, aloperine, and matrine) derived from *Sophora flavescens* Ait. and observed that sophocarpine demonstrated the most significant anti-inflammatory effect. Furthermore, they identified that sophocarpine mitigated DSS-induced colitis inflammation and intestinal fibrosis by modulating the SIRT 1/NF-κB p65 signaling pathway, reversing the senescence-associated secretory phenotype (SASP) and fibroblast-into-myofibroblast transition (FMT) of fibroblasts, as well as maintaining intestinal mucosal homeostasis (Jiang et al., 2024). In a rat model of 2,4,6-trinitrobenzene sulfonic acid-induced colitis, the total alkaloid *Sophora alopecuroides* (TASA), whose main ingredient is sophocarpine, was found to exert a strong anti-inflammatory effect by upregulating the levels of CD4^+^CD25^+^ Tregs and IL-10 in the colon and peripheral blood (Zhou et al., 2010a). Notably, sophocarpine has anti-asthmatic activity in an ovalbumin-induced mouse asthma model, as evidenced by the regulation of Th1/Th2 cytokine production, reduced pulmonary damage, inflammatory cell infiltration and decreased serum levels of IgE levels (Zhi et al., 2021). The aforementioned study suggested that sophocarpine exhibits remarkable efficacy in combating a wide range of inflammatory diseases, and is therefore a highly promising candidate for the development of novel anti-inflammatory strategies. However, it is worth noting that there is a dearth of pertinent research on the role of sophocarpine in other inflammatory conditions such as pneumonia, esophagitis, and gastritis. However, further investigations are needed to elucidate the pharmacological effects and intricate mechanisms of action underlying the effects of sophocarpine in these pathological conditions.

### Sophocarpine and virus

Sophocarpine reportedly has anti-hepatitis B virus (HBV) activity. A study comparing the anti-HBV effects of different alkaloids showed that HepG2.2.15 cells exposured to 0.4 or 1.6 mM sophocarpine had more effective decreases in hepatitis B surface antigen (HBsAg) levels in the medium than did those exposed to sophoridine or lamivudine, and sophocarpine produced the greatest reduction in HBsAg levels after 24 h of exposure. Moreover, sophocarpine concentration-dependently reduced the level of HBV DNA in culture media (Chen et al., 2016). Ding et al. isolated the ingredients of the roots of *Sophora flavescens* Ait. Using chromatography and found that sophocarpine was one of the major components. These authors further confirmed its anti-HBV activity, as evidenced by reduced HBsAg and hepatitis B “e” antigen (HBeAg) secretion in the HepG2 2.2.15 cell line (Ding et al., 2006). Liu et al. showed similar results that the co-administration of all four kinds of matrine type alkaloids (sophocarpine, oxymatrine, matrine, sophoridine) with thymopolypeptides could inhibit HBsAg and HBeAg secretion and HBV DNA replication in HepG2.2.15 cells (Liu et al., 2016). The studies mentioned above illustrate the significant anti-HBV activity of sophocarpine. However, these findings are currently limited to cell-based experimental models, thus, confirmation of the independent anti-HBV activity of sophocarpine in *in vivo* is still lacking. Furthermore, the specific molecular mechanisms through which sophocarpine counteracts HBV infection, including its potential involvement in established anti-HBV pathways such as liver X receptor pathways (Zeng et al., 2020), AMPK-ULK1 pathway (Wang et al., 2021d), and the cGAS-STING pathway (Zhao et al., 2023), have not been identified. Therefore, further investigations are warranted to address these pertinent issues.

Additionally, sophocarpine was found to attenuate liver injury in patients with concanavalin A-induced hepatitis. The protective effect of these agents was related to the inhibition of proinflammatory cytokines, chemokines, and the IFN-γ/STAT1 signaling pathway (Sang et al., 2017). Furthermore, N-substituted sophocarpinic acid derivatives, such as (*E*)-β, γ-*N*-(benzenesulfonyl) sophocarpinic acids, were shown to have anti-enteric activities against coxsackievirus. Notably, the inhibitory effect of (*E*)-12-*N*-(*m*-cyanobenzenesulfonyl)-β,γ-sophocarpinic acid against coxsackievirus B3 (CVB3) and coxsackievirus B6 (CVB6) in Vero cells is particularly noteworthy, and it can be safely administered orally, exhibiting an AUC value of 7.29 μM h. The maximum concentration (C_max_) in plasma was 4.54 μM, indicating rapid absorption with a maximum time (T_max_) of 0.5 h and a conducive half-time (t_1/2_) of 1.17 h. Additionally, the mean residence time (MRT) in rats was 1.5 h, and the LD50 in mice was found to exceed >1,000 mg kg^−1^ (Gao et al., 2013). Additionally, sophocarpine could inhibit human herpesvirus 6 (HHV-6) replication with selective indices of 184 and 183 (Qavi et al., 2002). Enterovirus 71 (EV71) is a major cause of hand, foot and mouth disease (HFMD) in children (Nayak et al., 2022). Jin’s group demonstrated that sophocarpine effectively inhibited the attachment and penetration of EV71, therefore preventing the entry of the virus into the cells. It also suppressed the replication of viral genomic RNA, suggesting that sophocarpine has anti-EV71 infection activity (Jin et al., 2017). Zhang et al. used a network pharmacology analysis technique followed by experimental validation and reported that sophocarpine may have potential therapeutic effects on coronavirus disease 2019 (COVID-19) by mediating cytokine release and the nuclear factor NF-κB signaling pathway (Zhang and Zhang, 2022). In brief, these reports indicate that sophocarpine may be a promising agent for the management of a wide range of viral infections.

### Sophocarpine and parasites

Parasitic diseases have long posed a substantial threat to public health, particularly in tropical regions where their prevalence remains alarmingly high. Consequently, there is an urgent need for novel antiparasitic strategies to effectively address the current situation (Pink et al., 2005). Cystic echinococcosis (CE) is a persistent parasitic affliction caused by the larval stage of *E. granulosus sensu lato*, which impacts both animals and humans alike. The ability of this disease to infect both human and livestock populations, coupled with the inadequate efficacy of existing therapeutic interventions, exacerbates its transmission dynamics while inflicting significant economic losses and compromising patient wellbeing. Luo et al. found that the water-soluble alkaloids E2-a from *Sophora moorcroftiana* (Benth.) Benth. ex Baker seeds could reduce cyst weight and stimulate a specific immune response targeting T cells in protoscolex-infected mice, suggesting that the E2-a fraction may be used as a potential therapeutic agent against *E. granulosus* infection (Luo et al., 2018). E2-a primarily comprises two crucial constituents, matrine and sophocarpine. However, there is currently no research validating the efficacy of either sophocarpine or matrine alone in combating *E. granulosus* infection. Furthermore, it remains unclear whether sophocarpine has therapeutic potential against other parasitic diseases such as leishmaniasis, filariasis, and malaria. In conclusion, sophocarpine represents a promising drug candidate that can serve as a key monomer or fundamental framework for developing novel antiparasitic drugs; hence, further investigations are necessary to elucidate its relevant pharmacological effects and mechanisms of action.

### Sophocarpine and cancer

The strong chemotherapeutic capacities of medicinal herbs and their derivative phytocompounds have been repeatedly confirmed by experimental and clinical studies on various cancer types (Huang et al., 2018). It can effectively improve the quality of life, survival and outcome of cancer patients (Luo et al., 2019). A large number of studies have confirmed the antitumor effect of sophocarpine. It may enhance antitumor immunity when used alone or in combination with other therapeutics. Based on the literature, sophocarpine has been shown to have anticancer effects on lung cancer, colorectal cancer, cervical cancer, head and neck cancer, prostate cancer, myeloma and liver cancer. In a study exploring the efficiency of sophocarpine in treating non-small cell lung cancer (NSCLC), the authors used a systems pharmacology and bioinformatics approach, in combination with C57/BL6 mice and different cell lines, including human NSCLC cell lines (NCI-H1975 and A549) and mouse Lewis lung carcinoma cell lines, and found that the combination of sophocarpine and an anti-PD-L1 antibody significantly inhibited tumor growth via a mechanism involving the ADORA1-ATF3-PD-L1 axis (Chen et al., 2022b). To evaluate the antitumor effects of different alkaloids, Lin et al. used human cancer cell lines of differing tissue origins. They found that sophocarpine (IC_50_: 3.68 mM) had a selective effect on different types of cancer, and had a significant inhibitory effect on lung cancer A549 cells (Lin et al., 2011). Moreover, sophocarpine was shown to inhibit colorectal cancer cell proliferation, invasion, and migration via a mechanism involving downregulation of the MEK/ERK/VEGF pathway, while overexpression of MEK reversed the beneficial effect of sophocarpine (Wang et al., 2019a). Meanwhile, the inhibitory effect of oxaliplatin on colorectal cancer liver metastasis could be further potentiated by the administration of sophocarpine. Yang et al. demonstrated that sophocarpine enhances the anti-proliferative, anti-invasion, and anti-migration effects of oxaliplatin on LoVo human colon cancer cells *in vitro*, and augments the inhibitory effect of oxaliplatin on nude mice with colon cancer liver metastasis (CCLM) *in vivo* (Yang et al., 2021c). Furthermore, sophocarpine dose-dependently inhibited the growth of the gastric cancer cell line MKN45 and BGC-823 by interrupting cell cycle progression, inhibiting proliferation and apoptosis. The mechanism underlying this effect was associated with autophagy induction and the regulation of the PTEN/PI3K/AKT pathway. However, unlike in previous studies of gastrointestinal tract cancer, no effect on inhibiting invasion or migration of cancer cells was mentioned, and whether the regulatory effect of p53, Bax, and Bcl-2 was related to the PTEN/PI3K/AKT pathway deserves further research (Huang et al., 2019).

Cancer cachexia is a multifactorial syndrome that leads to high morbidity and mortality in patients with advanced cancer. Zhang et al. tested the therapeutic effect of different kinds of alkaloids, including matrine, oxymatrine, sophocarpine, sophoramine, and sophoridine, on cachexia-related symptoms induced by colon-26 adenocarcinoma (C26). They found that sophocarpine exerted the most potent inhibitory effect on TNF-α and IL-6 production in both RAW264.7 cells and murine primary macrophages and had a better therapeutic effect on attenuating cachexia symptoms (Zhang et al., 2008). In addition, Li et al. extracted and purified total alkaloids from *Sophora alopecuroides* L. using macroporous resin and found that their active components, including sophocarpine, could effectively reduce the proliferation and apoptosis of human cervical tumor HeLa cells; However, it was not clear whether this effect occurred through the inhibition of the release of inflammatory factors by cancer cells or host cells (Li et al., 2016). In a study exploring the chemopreventive effect of sophocarpine, treatment of the head and neck squamous carcinoma cell lines UM-SCC-22B and UM-SCC-47 with sophocarpine resulted in a dose-dependent inhibition of proliferation, migration, and invasion. In addition, sophocarpine treatment activated p38 MAPK and repressed miR-21 expression by specifically blocking Dicer processing of premiR-21 to mature miR-21. Sophocarpine upregulated phosphatase and tensin homolog (PTEN), a target gene of miR-21, causing the inhibition of epithelial-mesenchymal transition in cancer cells. One study suggested that sophocarpine may be a potent miR-21 inhibitor for cancer treatment (Liu et al., 2017a). The antitumor effect of sophocarpine on prostate cancer was explored. Wang et al. used a comprehensive 2D PC-3 cell membrane chromatography (CMC) system to identify anti-prostate cancer components, including sophocarpine, and found that sophocarpine effectively inhibited epidermal growth factor-induced prostate cancer (PC-3) cell proliferation and induced apoptosis in a dose-dependent manner (Wang et al., 2017). Researchers further demonstrated that sophocarpine treatment suppressed the proliferation, migration and invasion of two castration-resistant prostate cancer cell lines, DU145 and PC-3 by deactivating the PI3K/AKT/mTOR signaling pathway (Weng et al., 2022a). In a study of myeloma, sophocarpine triflorohydrazone was found to inhibit KRASA12 and AMO-1 myeloma cell proliferation by promoting the expression of proapoptotic proteins and activation of Notch3-p53 signaling (Wang et al., 2021e). Consistently, research conducted by Zhang’s group tested the efficacy of sophocarpine against hepatoma cells and cancer stem cells. They found that sophocarpine exerts its antitumor effects by mediating the AKT/GSK3β/β-catenin axis and inhibiting epithelial-to-mesenchymal transition (EMT) induced by TGF-β (Zhang et al., 2016).

**FIGURE 2:**
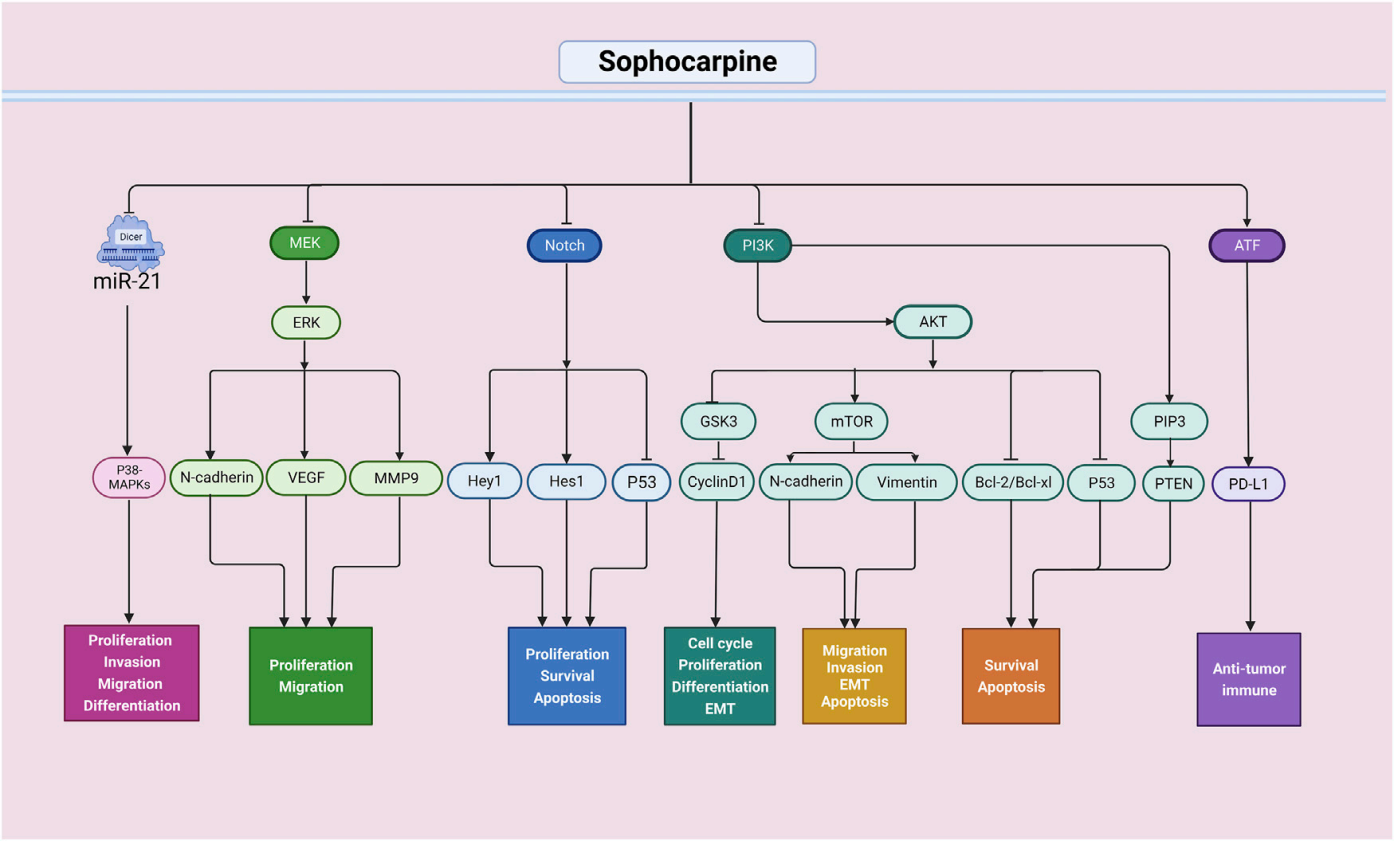
Antitumor effects of sophocarpine through multiple signaling pathways. The antitumor activity of sophocarpine is mainly achieved by interfering with multiple signaling pathways in tumor cells. It can inhibit the proliferation and invasion of tumor cells, as well as their migration and metastasis. Additionally, sophocarpine can regulate the apoptotic signaling pathway in tumor cells, promoting their apoptosis and thus inhibiting tumor growth and spread. Furthermore, sophocarpine also exhibited immunomodulatory effects on tumors. Overall, as a natural compound, sophocarpine has promising potential in the field of cancer treatment.

In summary, these basic studies have shown that sophocarpine has strong anticancer effects and thus may serve as a potential anticancer agent in clinical settings (Figure 2). Additionally, during development, sophocarpine can act against tumor cells by regulating proliferation, apoptosis, invasion, metastasis, and the tumor microenvironment, among other processes. However, most of those studies focused only on one aspect, and it is necessary to evaluate whether sophocarpine can exert multifaceted effects on various types of tumors.

### Sophocarpine and heart disease

Heart disease has emerged as a paramount global health concern, with its burden steadily escalating over the past decades. Based on pertinent research estimates, heart disease is projected to persist as the predominant contributor to the world’s disease burden in forthcoming decades (Roth et al., 2020). In recent years, natural agents have received increased amounts of attention as cardioprotective agents.

**FIGURE 3:**
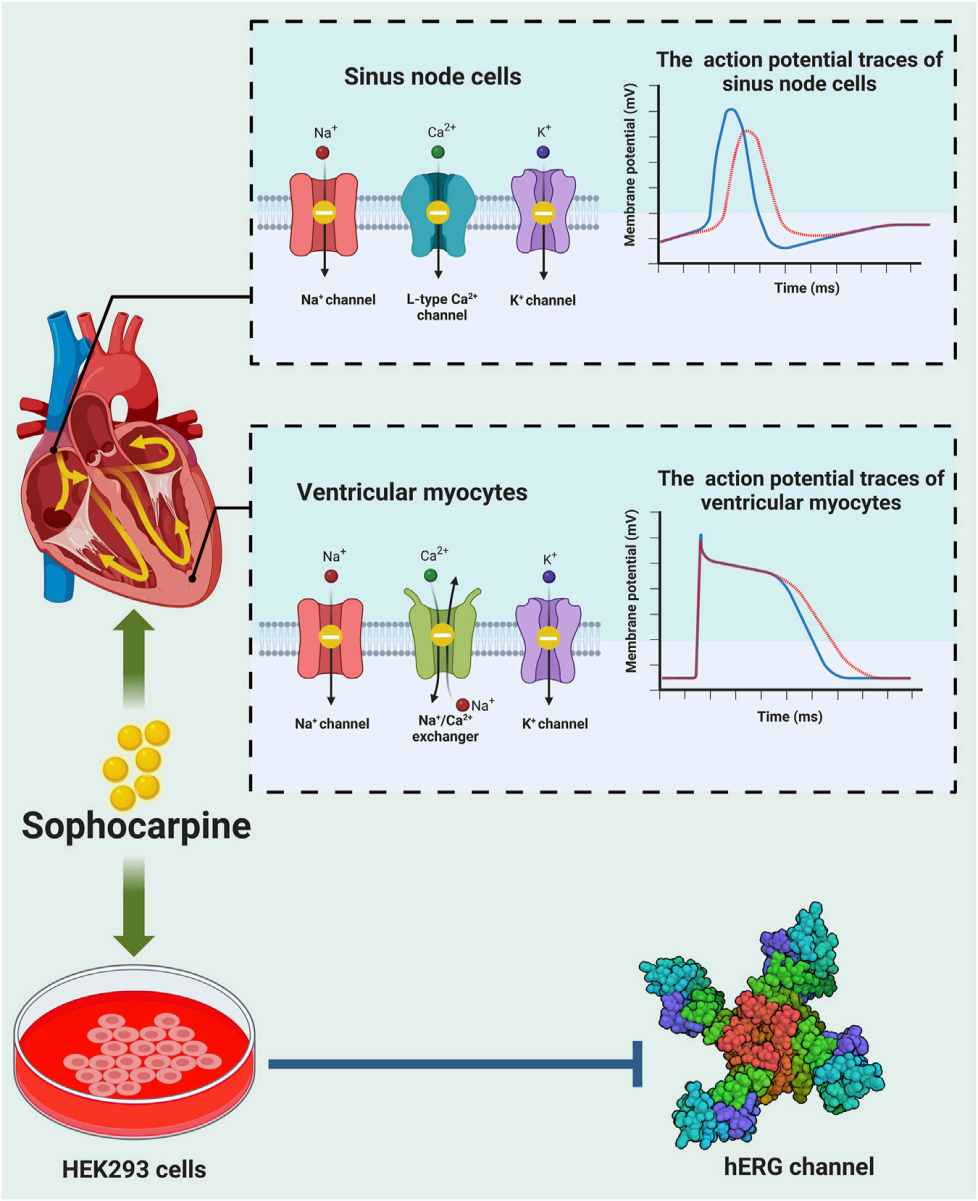
Anti-arrhythmic effects of sophocarpine. Sophocarpine has the property of blocks multiple ion channels, and its antiarrhythmic function is the result of its combined effect on these ion channels. *Top:* When sophocarpine was used to conduct action potential experiments on the sinoatrial node and ventricular muscles, it is found to significantly inhibit potassium, sodium, calcium ion channels and sodium-calcium exchangers (*left*), leading to a decrease in action potential (AP), prolongation of action potential duration (APD) and decrease in the action potential peak (*right*). blue strand: normal AP trace; red strand: AP trace after treatment with sophocarpine. *Bottom:* Sophocarpine can inhibit the hERG channel (PDB:7CN0) -mediated potassium ion tail current in HEK293 cells. These effects reverse the arrhythmias induced by isoproterenol.

Cardiac electrical activity is determined by different ion channels in cardiac myocytes. Cardiac arrhythmia occurs due to alterations in ion channel function (Katz, 1993). Sophocarpine was shown to have antiarrhythmic activity (Figure 3). It could prolong the action potential duration of guinea pig papillary muscle (Zhu et al., 1989). However, this study did not explore the potential mechanism by which sophocarpine exerts its antiarrhythmic effect through modulation of ion channel function. Subsequently, sophocarpine was found to inhibit late sodium current, the Na^+^/Ca^2+^ exchanger current, diastolic calcium concentration, and ventricular muscle contractility in rabbit ventricular myocytes (Zhang et al., 2012). Yang et al. further examined the electrophysiological effects of sophocarpine on cardiac channel currents and reported that sophocarpine was effective at modulating sodium, calcium and potassium channel currents (Yang et al., 2011). The human ether-a-go-go-related gene (hERG), also known as the KCNH2 gene, encodes the Kv11.1 protein, which serves as the α subunit of a voltage-sensitive potassium channel. The hERG channel plays a pivotal role in modulating the repolarization phase of cardiac action potentials by precisely regulating the rapid delayed rectifier K^+^ current (*I*_*kr*_). This regulation effectively influences both the action potential duration (APD) and QT interval observed via electrocardiograms, thereby impacting the occurrence of cardiac arrhythmias (Vandenberg et al., 2012; Butler et al., 2019). Using the whole-cell patch-clamp technique, Qi et al. found that sophocarpine inhibited transfected human ether-a-go-go-related gene (hERG) channels in a concentration-dependent manner by influencing the inactivation state (Qi et al., 2008). In addition, sophocarpine had no effect on the generation and trafficking of the hERG protein (Zhao et al., 2008). These authors further compared the effects of sophocarpine and sophoridine on hERG channels and showed that sophocarpine acted as a more potent hERG K^+^ channel blocker than sophoridine (Zhao et al., 2009). These studies consistently demonstrated that sophocarpine exerts antiarrhythmic effects through the inhibition of the hERG channel. However, further investigates of alternative cell types, such as cardiac myocytes or *in vitro* animal tissues, and comparisons of the findings with those obtained from HEK293 cells are needed to enhance the scientific rigor and broaden the scope of this research. In addition, aloperine, another alkaloid derived from *Sophora flavescens* Ait., has been scientifically proven to possess potent antiarrhythmic effects (Hu et al., 2023) and to act as a natural KCNQ5 agonist (Manville et al., 2019). Hence, it is intriguing to explore whether sophocarpine also modulates other currents such as the slow delayed rectifier K^+^ current (*I*_*ks*_), transient outward K^+^ current (*I*_*to*_), or other α subunits, such as the KCNQ1 gene and its upstream regulatory genes of the KCNE family, in addition to regulating IKr currents associated with the hERG. Moreover, conducting further *in vivo* experiments utilizing appropriate animal models such as mice or rats would be imperative for validating the antiarrhythmic resistance effect of sophocarpine.

Moreover, in a rat model of myocardial ischemia and reperfusion injury, sophocarpine was found to limit infarct size post-I/R, accompanied by decreased serum levels of TNF-α, IL-6, IL-10, and myeloperoxidase (MPO) activity, indicating reduced inflammation. The authors further proved that NF-κB inactivation may play a role in sophocarpine-induced cardioprotection (Li et al., 2011). Additionally, Zhang et al. conducted a study on the therapeutic potential of sophocarpine in mitigating doxorubicin-induced heart injury in mice and H9C2 cells. Their findings demonstrated that sophocarpine effectively ameliorated doxorubicin-induced cardiac dysfunction, while concurrently reducing levels of creatine kinase (CK), creatine kinase-MB (CK-MB), lactate dehydrogenase (LDH), as well as oxidative stress markers such as malondialdehyde (MDA), superoxide dismutase (SOD), and glutathione (GSH) in mouse serum. Furthermore, sophocarpine was shown to decrease apoptosis and oxidative stress levels in both mice and H9C2 cells through activation of the Nrf 2/HO-1 signaling pathway at a molecular level (Zhang et al., 2024). Similarly, in an overload-induced cardiac fibrosis rat model, sophocarpine was effective at attenuating cardiac fibrosis, as indicated by reduced heart weight to body weight ratio and improved hemodynamics. It decreases the levels of proinflammatory cytokines, collagen content, and matrix metalloproteinases (MMPs) by modulating the NF-κB signaling pathway (Li et al., 2014). It has been shown that sophocarpine can alleviate sepsis-induced cardiomyopathy. It effectively inhibited LPS-induced cardiac inflammation, oxidative stress, apoptosis, and autophagy. A follow-up mechanistic study suggested that sophocarpine protects cells by inhibiting TLR-4/NF-κB while activating the Nrf2/HO-1 signaling pathway (Fu et al., 2023). These studies provide evidence that sophocarpine has cardioprotective effects on diverse forms of damage by mitigating inflammation, attenuating oxidative stress, and engaging multiple signaling pathways and mechanisms. Further investigation is warranted to elucidate the therapeutic and protective effects of sophocarpine on other types of heart diseases such as myocardial hypertrophy and ventricular remodeling, as well as to uncover the underlying mechanisms of action.

Taken together, these studies not only identified sophocarpine as a potential antiarrhythmic agent but also provided evidence for its potential therapeutic value in treating cardiac fibrosis as well as different types of cardiac injury.

### Sophocarpine and vascular disease

The accumulation of oxidized low-density lipoprotein (ox-LDL) leads to endothelial cell injury and has been recognized as the major factor in the development of arterial atherosclerosis (Poznyak et al., 2020). Sophocarpine was found to attenuate ox-LDL-induced endothelial cell dysfunction and apoptosis through suppression of the NF-κB pathway in human aortic endothelial cells (Fang et al., 2020). Furthermore, sophocarpine could produce endothelial protective effects against advanced glycation end product-induced reactive oxygen species-mediated apoptosis by targeting MKK3 and p38MAPK signaling (Liu et al., 2017b). Balloon angioplasty is a general interventional technique applied for treating coronary arteries and endovascular vessel-related disease. However, in-stent restenosis often occurs and may lead to prolonged hospitalization and increased mortality (Katsanos et al., 2018). In a rat model, Yang et al. reported that sophocarpine effectively alleviated intimal hyperplasia following balloon injury via the modulation of inflammation-related signals (Yang et al., 2021a). In addition, sophocarpine alleviated the restenosis induced by cigarette smoke in rats postangioplasty (Yang et al., 2020). These studies support the idea that sophocarpine is capable of reducing endothelial cell injury and maintaining vessel integrity and therefore may be used to treat atherosclerosis or vessel dysfunction after balloon angioplasty. However, the role of sophocarpine in hypertension, a prevalent chronic disease, has not been fully elucidated. Conversely, extensive research has demonstrated the vasodilatory effects and reduction in vascular pressure associated with matrine and aloperine extracted from *Sophora flavescens* Ait. (Zheng et al., 2009; Shan et al., 2023). Given that sophocarpine can regulate multiple ion channels and safeguard vascular endothelium, it unequivocally holds potential for antihypertensive effects. Nevertheless, further investigation is imperative to substantiate this assertion.

### Hepatic protective activity

Sophocarpine was shown to have a hepatoprotective effect on different types of liver injury. It was shown to attenuate hepatic oxidative reactions, inflammation, and apoptosis induced by lipopolysaccharide via a mechanism related to suppressing the CYP2E/Nrf2/ROS and PI3K/AKT pathways (Zhengyu et al., 2018). In a mouse cecal ligation and puncture model, sophocarpine alleviated septic liver injury, as evidenced by reduced serum levels of aspartate aminotransferase (AST) and alanine aminotransferase (ALT) and inflammatory responses. Sophocarpine-induced the nucleotide-binding oligomerization domain, leucine rich repeat and pyrin domain containing 3 (NLRP3) degradation by autophagy may be responsible for its protective effects (Hou et al., 2020). The above two studies employed distinct murine models of septic liver injury, providing ample evidence supporting the protective effect of sophocarpine against this injury through multiple mechanisms. Another study conducted by Huang et al. showed that sophocarpine could inhibit NK cell activity and thus attenuate the immunological liver injury induced by Poly I:C/D-GalN in C57BL/6 mice (Huang et al., 2016). Further investigations into the regulatory effects of sophocarpine on other immune cells, including T cells and macrophages, are important for elucidating the role of sophocarpine.

Using two types of injury models (dimethylnitrosamine injection and bile duct ligation), sophocarpine was found to suppress hepatic fibrosis and inhibit the activation and proliferation of hepatic stellate cells (Qian et al., 2014). Additionally, sophocarpine also has an anti-nonalcoholic steatohepatitis effect (Song et al., 2011), and the mechanism may involve the activation of the AMPK signaling pathway (Song et al., 2013). Later, the same group further demonstrated that sophocarpine treatment reduced Toll-like receptor 4 expression levels and suppressed proinflammatory cytokine synthesis in oleic acid-induced steatotic hepatocytes (Song et al., 2015).

### Neuroprotective activity

Using a rat model of transient focal cerebral ischemia, sophocarpine was shown to ameliorate brain damage, as indicated by reduced infarction and apoptosis, accompanied by improved neurological scores. The observed positive outcomes could potentially be attributed to the inhibitory action on acid-sensing ion channel 1 (ASIC1) and potent anti-apoptotic effects exerted by sophocarpine, thereby suggesting a plausible mechanism underlying its beneficial effects (Yifeng et al., 2011). Zhu et al. used β-amyloid to induce PC12 neuronal cell damage and evaluated the protective effect of sophocarpine. The authors found that the favorable effect of sophocarpine was associated with inhibition of NF-κB nuclear translocation (Zhu et al., 2021). The role of sophocarpine in improving neurological functions and cognitive performance was studied by Ye et al. They found that sophocarpine alleviated cognitive impairment and reduced neural loss via modulation of the inflammatory pathway in a mouse model of Alzheimer’s disease (Ye et al., 2021). The next step in research may involve the assessment of sophocarpine across various dosages and administration routes to investigate its potential role in the treatment of Alzheimer’s disease. In conclusion, despite the limitations of the current research, it is indisputable that sophocarpine will exhibit neuroprotective effects and hold promise as a novel therapeutic agent for major neurological disorders such as cerebral ischemia and Alzheimer’s disease.

### Pulmonary protective activity

The pulmonary protective effects of sophocarpine on acute lung injury (ALI) was evaluated in two studies. Han et al. used a mouse model of LPS-induced ALI and reported that sophocarpine treatment reduced the lung wet/dry ratio, pathological changes, and inflammatory responses (Han et al., 2006). The same results were obtained in another study carried out by Lu et al., who similarly evaluated the protective effect of sophocarpine in a mouse model change and additionally discovered sophocarpine decreased myeloperoxidase (MPO) activity and malondialdehyde (MDA) content. The underlying mechanism may be attributed to the modulation of TLR4 expression and NF-κB and MAPK activation (Lu et al., 2019). Patients suffering acute lung injury have high morbidity and mortality rates. Its pathogenesis is unclear, and treatment options are limited. Although the pulmonary protective effect of sophocarpine was confirmed, whether sophocarpine is still protective against other types of ALI, such as transfusion-, pulmonary ischemia/reperfusion injury-, or ventilator-induced ALI, would be interesting topics in the future.

### Renal protective activity

It has been shown that in a mouse model of isoproterenol (ISO)-induced kidney injury, sophocarpine could alleviate kidney injury by reducing the serum levels of serum creatinine (SCr) and blood urea nitrogen (BUN), inhibiting inflammatory cytokine release, preventing fibrosis, and reducing apoptosis and oxidative stress. The TLR-4/NF-κB, TGF-β1/Smad3, and Nrf2/HO-1 signaling pathways may participate in sophocarpine-mediated renal protection (Zhou et al., 2022). Lupus nephritis is a complication of the autoimmune disease systemic lupus erythematosus (Anders et al., 2020). Sophocarpine effectively protected kidneys against lupus nephritis in MRL/lpr mice. It reduced the renal inflammatory response and inhibited NLRP3 inflammasome activation. The underlying mechanism may be associated with the IKKS-NF-κB pathway (Li et al., 2018). Although information regarding sophocarpine-induced renal protection is limited, these studies may shed light on novel therapeutic options for treating kidney disease with sophocarpine.

### Sophocarpine and morphine withdrawal syndrome

The effect of *Sophora alopecuroides* var. *alopecuroides* seeds on morphine withdrawal syndrome was evaluated. The authors compared the pharmacological effects of the seed total extract, alkaloid fraction and major compound matrine to those of saline and methadone on mice with morphine withdrawal syndrome and found that all the abovementioned reagents decreased jumping and diarrhea. They further used gas chromatography‒mass spectrometry to analyze the main ingredients involved in the extraction of alkaloids and found that sophocarpine is one of the major constituents (Kianbakht and Hashem Dabaghian, 2016). The same group subsequently conducted a randomized, double-blind, and placebo-controlled clinical trial on abstinent opium addicts who took alkaloid components of *S. alopecuroides* extract capsules once daily for 8 consecutive days. The clinical opiate withdrawal scale (COWS) was used to assess withdrawal symptoms. Patients treated with extract capsules had lower COWS scores than did those in the placebo group. Moreover, it did not affect blood parameters. The extract may be a potential therapeutic agent for acute opioid withdrawal symptoms (Kianbakht et al., 2020). The utilization of traditional Chinese herbal extracts in the management of opiate withdrawal syndrome represents a pioneering and noteworthy area of research. Future investigations should further elucidate the potential underlying mechanisms of action of these compounds, as well as explore the therapeutic effects of specific compounds such as matrine and sophocarpine on opiate withdrawal syndrome. It is crucial to investigate whether these effects are associated with μ, κ, and δ opioid receptors or nicotinic receptors (Rahman et al., 2014). Furthermore, considering the global prevalence of methamphetamine addiction and alcohol dependence, it is worthwhile to further explore whether *S. alopecuroides* extract or sophocarpine could serve as treatment options for these conditions.

### Sophocarpine and diabetes

Su et al. tested the antidiabetic effect of sophocarpine in a mouse model of streptozotocin (STZ)-induced type 2 diabetes. They found that sophocarpine regulated blood glucose, insulin, LDL-c content and HDL-c levels while concurrently mitigating pancreatic injury and effectively impeding the advancement of type 2 diabetes by attenuating peroxisome proliferator-activated receptor gamma (PPARγ) activity in mice with type 2 diabetes (Su et al., 2021). Although sophocarpine was shown to have beneficial effects in this study, further animal and human studies are needed to confirm and evaluate the role of sophocarpine in both type 1 and type 2 diabetes.

### Sophocarpine and analgesia

Using different mouse models, such as carrageenan-induced rat hind paw edema, xylene-induced mouse ear edema and acetic acid-induced mouse vascular permeation, sophocarpine was found to possess analgesic and anti-inflammatory properties (Gao et al., 2009) and anti-inflammatory activities via the possible mechanism of affecting Ca^2+^ influx and inhibiting the secretion of inflammatory factors (Wang et al., 2021b). The effect of sophocarpine on neuropathic pain induced by chronic constriction injury was further evaluated by Jin et al. These authors showed that sophocarpine was effective at increasing the mechanical withdrawal threshold (MWT), thermal withdrawal latency (TWL), and tail-curling latency while reducing the cold withdrawal threshold (CWT) (Jin et al., 2019). The pharmacological effects and mechanism of action of sophocarpine are listed in Table 2.

**TABLE 2:** Pharmacological effects of sophocarpine

Pharmacological effects	Disease	Cell lines/Animal	Forms/Dose	Source	Result and mechanism	References
Anti-inflammation	Rheumatoid arthritis	Human RA-FLS cell lines	Sophocarpine, 0.2,0.5,1,2 mg/mL	Not mentioned	Inhibited the expression of proinflammatory genes, downregulated LPS-induced NF-kB and MAPK signaling in human RA-FLS cell lines, suppressed the development of collagen-induced arthritis	Zhu and Zhu (2017)
	Osteoarthritis	Rat, rat chondrocytes	Sophocarpine, 10 mg/kg, 50,100,200 μM	Purchased, purity ≥98%	Protected chondrocytes from IL-1β-induced ECM degradation, inhibited the production of inflammatory mediators, inhibited NF-κB activation, regulated the PI3K/AKT signaling pathway in IL-1β-stimulated chondrocytes, alleviated the development of osteoarthritis in a rat destabilization of the medial meniscus model	Wu et al. (2019)
	LPS-induced inflammatory responses	RAW 264.7	Sophocarpine, 25,50,100 μg/mL	Extracted and isolated from *Sophora alopecuroides* L.	Decreased COX-2, TNF-α and IL-6 expressions, NO production and iNOS expression, prevented NF-κB transcriptional activity, inhibited phosphorylation of IκB-α, suppressed MAPK signaling pathway activation	Gao et al. (2012)
	Anti-inflammatory tests	RAW 264.7, zebrafish	Sophocarpine, 50 μM	Extracted and isolated from the roots of *Sophora tonkinensis* Gagnep.	Reduced the levels of IL-6 and TNF-α in RAW264.7, reduced the migration of neutrophils in zebrafish	He et al. (2019)
	Osteolytic diseases	Rat, bone marrow-derived macrophage	Sophocarpine, 20 mg/kg, 0.31–2 mM	Purchased	Suppressed the receptor activator of NF-κB ligand (RANKL)-induced osteoclastogenesis, attenuated the formation of F-actin ring and bone resorption and downregulated osteoclast-specific gene expression levels, inhibited rat osteoclast formation by suppressing activation of the NF-κB signaling pathway *in vitro*, inhibited implant loosening, osteoclastogenesis and serum marker of bone resorption, attenuated implant loosening through NF-κB signaling *in vivo*	Zhou et al. (2018)
	DSS-induced colitis	Mice	Sophocarpine, 15,30,60 mg/kg	Purchased, purity ≥98%	Reduced disease activity index and wet weight of colons, decreased myeloperoxidase activity and the level of IL-1 and IL-6 in serum	Wang et al. (2012)
	DSS-induced colitis	Mice	Sophocarpine, 30 mg/kg	Purchased	Reduced disease activity index, TNF-α, IL-1β, IL-6 and inflammatory reactions, inhibited TLR4/MAPKs, K2/STAT3 signaling pathway activation	Zhang et al. (2015)
	DSS-induced colitis	Mice, HIF cells	Sophocarpine, 30, 60 mg/kg, 40, 80, and 160 μM	Purchased, purity ≥98%	Improved disease symptoms and histopathological features, reduced the release of pro-inflammatory cytokines TNF-α, IL-6, IL-1β, decreased the expression of fibrotic biomarkers α-Sma, vimentin, and p65 protein, reversed the SASP and FMT of fibroblasts, SIRT 1 is activated to inhibit the activation of the p65 pathway, regulated the NF-κB/STAT 3 cell signaling pathways	Jiang et al. (2024)
	Colitis	Rat	Total alkaloids of S*ophora alopecuroide*s (TASA), 15,30,60 mg/kg	Extracted and isolated from S*ophora alopecuroides* L.	Upregulated CD4^+^CD25^+^Tregs and IL-10, decreased the DAI and histological grading of colitis	Zhou et al. (2010a)
	Asthma	Mice	Sophocarpine, 10,20,40 mg/kg	Purchased, purity ≥98%	Alleviated pulmonary damage, regulated Th1/Th2 cytokines production (IL-4, IL-5 and INF-γ) in broncho-alveolar lavage fluid, reduced IgE level in serum, inhibited inflammatory cell infiltration; proteomic results showed that binding energy of 87 targets was varied from −9.72 kcal/mol to 227.16 kcal/mol. It suppressed arrb2, ANXA1, MYD88 and SPHK1 expression and activated p-STAT1	Zhi et al. (2021)
Anti-virus	HBV	HepG2.2.15	Sophocarpine, 0.4–1.6 mM	Purchased, purity ≥98%	Reduced the HBsAg, HBeAg and HBV DNA level	Chen et al. (2016)
	HBV	HepG2.2.15	Sophocarpine, 0.1–0.2 μmol/L	Extracted and isolated from the roots of *Sophora flavescens* Ait.	Reduced HBsAg and HBeAg secretion	Ding et al. (2006)
	HBV	HepG2.2.15	Sophocarpine combined with thymopolypeptides, 0.2–6.4 mM	Purchased, purity ≥98%	Reduced the HBsAg, HBeAg and HBV DNA level of the medium	Liu et al. (2016)
	Concanavalin A-induced hepatitis	Mice	Sophocarpine, 30,60 mg/kg	Not mentioned	Ameliorated liver inflammation and injury, reduced RNA expression levels of chemokines and adhesion molecules, mediated IFN-γ/STAT1 signaling pathway	Sang et al. (2017)
	Coxsackievirus	Vero cells	(*E*)-12-*N*-(*m*-cyanobenzenesulfonyl)-β,γ-sophocarpinic acid, 0–1,000 mg/kg	Synthesis	Have anti-enteroviral activities against coxsackievirus, especially CVB3 and CVB6	Gao et al. (2013)
	HHV-6	HHV-6 Z29 strain, Molt-3 cells	Sophocarpine, 5 mg/mL	Extracted and isolated from *Sophora nuttalliana* B.L.Turner	Inhibited HHV-6 replication, selective indices (SI) values were 183 and 184	Qavi et al. (2002)
	Enterovirus 71	EV71, Vero cells	Sophocarpine, 15.625–4,000 μg/mL	Purchased	Inhibited the attachment and penetration of EV71, suppressed the replication of viral genomic RNA	Jin et al. (2017)
	COVID-19	Network pharmacology analysis	Sophocarpine	Purchased, purity ≥98%	Mediated the cytokine release and NF-κB signaling pathway	Zhang and Zhang (2022)
Anti-parasite	Protoscoleces infection	Mice	Low polarity compounds, 0.5 mg/mL	Extracted and isolated from *Sophora moorcroftiana (Benth.)* Benth. ex Baker seeds	Increased the frequency of CD3^+^CD4^+^ T and reduced the frequency of PD-1+ T cells in protoscolex-infected mice, increased Th1-, Th2-, Th9- and Th17-Type cytokines in culture supernatant of splenocytes	Luo et al. (2018)
Antitumor	Non-small cell lung cancer	NCI-H1975, A549, mouse Lewis lung carcinoma (LLC) cell lines, C57BL/6 mice	Combination of sophocarpine and anti-PD-L1 antibody, 2 mM, 5 mg/kg	Extracted and isolated from *Sophora alopecuroides* L.	Enhanced the efficacy of anti-PD-L1 immunotherapy, promoted PD-L1 expression via the ADORA1-ATF3 axis	Chen et al. (2022b)
	Multiple cancer types	HL-60, U937, K562, EC109 cells, A549 cells, HepG2 cell lines	Sophocarpine,1–20 mM	Purchased, purity ≥98%	Lung cancer A549 cells were sensitive to sophocarpine with IC_50_ close to 4 mM	Lin et al. (2011)
	Gastric cancer	Mice, MNK-45 and BGC-823 cell lines	Sophocarpine, 0–8 mg/mL, 50 mg/kg	Purchased	Inhibited growth, regulated cell autophagy in MKN45 and BGC-823 cell lines, caused cell apoptosis and cell cycle arrest in G0/G1 phase, suppressed PI3K/AKT signaling pathway	Huang et al. (2019)
	Colorectal cancer	Human colon cancer HCT116 and SW620 cell lines	Sophocarpine, 0–1.6 mM	Not mentioned	Suppressed the proliferation, inhibited the migration of HCT116 and SW620 cells, inhibited the level of VEGF family cytokines in cell supernatant. The mechanism involving the inhibition of MEK/ERK/VEGF pathway	Wang et al. (2019a)
	Colorectal cancer	Human colon cancer cell lines (LoVo)	Sophocarpine, 5.10 μmol/L, 3.57 mg/kg	Purchased	Enhanced the anti-proliferation, anti-invasion and anti-migration of oxaliplatin on LoVo cells, enhanced the inhibitory effect of oxaliplatin on colon cancer liver metastasis *in vivo*, inhibited EMT *in vitro* and *in vivo*	Yang et al. (2021c)
	Cancer cachexia	RAW264.7, mouse macrophages, colon-26 adenocarcinoma cells, mice	Sophocarpine, 0.5 mg/mL, 50 mg/kg	Purchased, purity ≥98%	Inhibited TNF-α and IL-6 production and expression in both RAW264.7 cells and murine primary macrophages, attenuated cachexia symptoms	Zhang et al. (2008)
	Cervical cancer	Human cervical tumor HeLa cells	Extracts of S*ophora alopecuroides* L., 6.25,8.75,12.50 mg/mL	Extracted and isolated from *Sophora alopecuroides* L.	Inhibited cell growth, induced apoptosis in cervical tumor HeLa cell	Li et al. (2016)
	Head and neck cancer	UM-SCC-22B cells, UM-SCC-47 cells, mice	Sophocarpine, 1,2,4 μM, 5 mg/kg	Purchased, purity ≥98%	Inhibited HNSCC cell proliferation, invasion, and migration through the p38 MAPK signaling pathway, upregulated PTEN, downregulated miR-21 by Blocking Dicer Processing, inhibited epithelial mesenchymal transition by regulating miR-21 expression	Liu et al. (2017a)
	Prostate cancer	PC-3 prostate cancer cells	Sophocarpine, 0.1–2 mg/mL	Purchased, purity ≥98%	Inhibited proliferation and induced apoptosis in PC-3 cells in a dose-dependent manner	Wang et al. (2017)
	Prostate cancer	DU145 and PC3 prostate cancer cell lines, mice	Sophocarpine, 0–225 mM, 35 mg/kg	Purchased, purity ≥98%	Inhibited the proliferation migration and invasion, induced apoptosis of CRPC cells, suppressed the epithelial-mesenchymal transition process, inactivated the PI3K/AKT/mTOR signaling pathway; inhibited tumor growth *in vivo*	Weng et al. (2022a)
	Myeloma	KRASA12 and AMO-1 cells	Sophocarpine triflorohydrazone, 21 μM	Synthesis	Reduced KRASA12 and AMO-1 cell viability, increased cell apoptosis, promoted HES1, p53 and HEY1 mRNA expression, decreased Notch3 protein expression	Wang et al. (2021e)
	Liver cancer	Human HCC-LM3 and MHCC-97H cell lines, mice	Sophocarpine, 10 mmol/L, 10 mg/mL	Purchased	Inhibited tumor growth, suppressed cell proliferation, blocked cell cycle was in the G0/G1 phase, reduced the number of cancer stem cells, reversed hepatocellular carcinoma malignant phenotype, downregulated the activity of the AKT/GSK-3β/β-catenin axis, inhibits epithelial to mesenchymal transition induced by TGF-β	Zhang et al. (2016)
Heart disease	Cardiac arrhythmia	Guinea pig papillary muscle	Sophocarpine, 50 µM	Not mentioned	Prolonged APD50, APD90 and ERP, unchanged ERP/APD90 ratio	Zhu et al. (1989)
	Cardiac arrhythmia	Rabbit ventricular myocytes	Sophocarpine, 20,40,80 μM	Purchased, purity ≥98%	Inhibited INa.L, INCX, diastolic Ca^2+^ concentration, and contractility in rabbit ventricular myocytes	Zhang et al. (2012)
	Cardiac arrhythmia	Guinea pig papillary muscle or rabbit sinus node cells	Sophocarpine, 15,300 μM	Purchased, purity ≥98%	Prevented tachyarrhythmia produced by isoprenaline, decrease the amplitude and maximal depolarization velocity of the fAP and Na^+^ current, prolonged the effective refractory period, decreased the amplitude and V_max_ of the sAP, attenuated the Ca^2+^ current the K^+^ tail current	Yang et al. (2011)
	Cardiac arrhythmia	HEK293 cells	Sophocarpine, 10,30,100,300 µM	Purchased, purity ≥98%	Inhibited hERG channels in a concentration-dependent manner, had no effect on channel activation and deactivation and the expression of HERG protein	(Qi et al., 2008)
	Cardiac arrhythmia	HEK293 cells	Sophocarpine, 10,30,100,300 μM	Purchased	Inhibited hERG, accelerated the time constants of inactivation, recovery from inactivation and onset of inactivation	Zhao et al. (2008)
	Cardiac arrhythmia	HEK293 cells	Sophocarpine, 10,30,100,300 µM	Purchased	More potent than sophoridine in inhibiting hERG tail current, a higher binding affinity for the inactivate state, no effect on the generation and trafficking of hERG protein	Zhao et al. (2009)
	Myocardial ischemia and reperfusion injury	Rat	Sophocarpine, 7.5, 15, 30 mg/kg	Extracted and isolated from *Sophora alopecuroides* L.	Reduced TNF-α, IL-6 and IL-10 levels and MPO activity, inhibited translocation of NF-κB, P38 and JNK phosphorylation	Li et al. (2011)
	Doxorubicin-induced heart injury	Mice, H9C2 cells	Sophocarpine, 10,30 mg/kg, 1, 2, 5 μM	Purchased, purity ≥98%	Increasing the left ventricle ejection fraction and the left ventricle fractional shortening, reduced serum markers of myocardial injury and oxidative stress, decreased the levels of pro-oxidative protein NOX-4 and apoptosis-related proteins Bax, cleaved-caspase 3 and cytochrome-c (Cyto-C), elevated the levels of antioxidant protein SOD-2 and anti-apoptotic protein Bcl-2 while activating the Nrf 2/HO-1 signaling pathway	Zhang et al. (2024)
	Cardiac fibrosis	Rat	Sophocarpine, 10, 20, 40 mg/kg	Extracted and isolated from *Sophora alopecuroides* L.	Decreased heart weight, improved hemodynamics, attenuated cardiac fibrosis, decreased pro-inflammatory cytokine levels and MMP-2, 9 expression, inhibited IκB-α phosphorylation	Li et al. (2014)
	Sepsis-induced cardiomyopathy	Mice, H9C2 cells	Sophocarpine, 20 mg/kg, 1, 5, 10 μM	Purchased, purity ≥98%	Reduced LPS-stimulated cardiac dysfunction and decreased cardiac apoptosis, inflammation, oxidative stress, reduced cardiac autophagy in mice and H9C2 cells	Fu et al. (2023)
Vascular disease	Atherosclerosis	Human aortic endothelial cells	Sophocarpine, 25–800 μg/mL	Purchased	Ameliorated ox-LDL-mediated HAECs cytotoxicity, DNA fragmentation, and apoptosis, downregulated the expression levels of pro-inflammatory mediators (TGF-β, IL-6, IL-1β, TNF-α) and pro-inflammatory vascular adhesion molecules (VCAM-1, ICAM-1, and E-selectin), through suppression of NF-κB signaling	Fang et al. (2020)
	Endothelial apoptosis	Rat aorta and cultured rat aortic endothelial cells	Sophocarpine, 40 mg/kg	Purchased	Alleviated advanced glycation end products (AGEs)-induced ROS generation, cell apoptosis in rat aorta and cultured RAECs, facilitated Nrf2 nuclear translocation and ARE-binding, recovered activation of MKK3/6-p38 MAPK/Nrf2 signaling	Liu et al. (2017b)
	Restenosis after angioplasty	Rat	Sophocarpine, 40 mg/kg	Purchased	Reduced the neointima to media ratio, decreased the protein levels of IL-6, IL-1β, MCP-1, NF-κB, TNF-α, ICAM-1 and VCAM-1, elevated eNOS levels	Yang et al. (2021a)
	Restenosis after angioplasty	Rat	Sophocarpine, 40 mg/kg	Purchased	Reduced the neointima to media ratio, inhibited the expression levels of Phospho-MKK3/6, Phospho-p38, IL-1β and TNF-α	Yang et al. (2020)
Hepatic protection	Septic liver injury	Mice, hepatic stellate cells	Sophocarpine, 5 mg/kg,1,2 μM	Not mentioned	Ameliorated hepatic oxidative stress, enhanced the expression of antioxidant molecules, attenuated regional and systematic inflammation, reduced apoptosis of hepatocytes, suppressed the CYP2E/Nrf2/ROS as well as PI3K/AKT pathways, inactivated p38/JNK cascade and NF- κB pathway	Zhengyu et al. (2018)
	Septic liver injury	Mice	Sophocarpine, 30,60 mg/kg	Not mentioned	Reduced serum levels of AST, ALT, IL-6 and IL-1β, suppressed liver IL-1β, NLRP3, caspase 1-p20 and gasdermin D-p30 protein levels, promoted the autophagy process	Hou et al. (2020)
	Immunological liver injury	Mice	Sophocarpine, 60,120 mg/kg	Purchased	Decreased the production of pro-inflammatory cytokines, suppressed NK cell activation and downregulated the expression of NKG2D, decreased the expression levels of DAP12, ZAP76 and SYK	Huang et al. (2016)
	Hepatic fibrosis	Rat, hepatic stellate cells	Sophocarpine, 20 mg/kg	Purchased, purity ≥98%	Decreased serum levels of aminotransferases, total bilirubin, prevented hepatic fibrosis, decreased the expression levels of pro-fibrotic cytokines and TLR4 signaling pathway-related proteins; inhibited the activation and proliferation of HSCs	Qian et al. (2014)
	Nonalcoholic steatohepatitis	Rat	Sophocarpine, 20 mg/kg	Purchased	Decreased liver weight, liver index, serum transaminase and serum lipids; reduced synthesis of inflammatory cytokines TNF-α, TGF-β1 and IL-6, activated protective adipocytokine adiponectin	Song et al. (2011)
	Nonalcoholic steatohepatitis	Rat hepatocytes	Sophocarpine, 0.2,0.4,0.8 mmol/L	Purchased	Alleviated the steatosis of primary hepatocytes, increased adiponectin expression levels, decreased leptin transcription levels, activated AMPK signaling pathway	Song et al. (2013)
	Nonalcoholic steatohepatitis	Rat hepatocytes	Sophocarpine, 0.2,0.4,0.8 mmol/L	Purchased, purity ≥98%	Suppressed pro-inflammatory cytokines synthesis and reduced the expression of TLR4, inhibited NF-κB, JNK, and ERK expression	Song et al. (2015)
Neuroprotection	Focal cerebral ischemia	Rat	Sophocarpine, 5,10,20 mg/kg	Purchased, purity ≥98%	Reduced infarction, improved neurological score, reduced apoptosis, downregulated the expression of acid-sensing ion channel 1 (ASIC1)	Yifeng et al. (2011)
	β-amyloid induced PC12 neuronal cell damage	PC12 cells	Sophocarpine, 0.25,0.5,0.75,1.0,1.5,2.0 μM	Purchased	Reversed suppressive effect of β-amyloid on PC12 cell growth, reduced PGE2, COX-2 expression and NF-κB nuclear translocation, attenuated iNOS Synthesis, prevented excessive NO production	Zhu et al. (2021)
	Alzheimer’s disease	Mice	Sophocarpine, 100 mg/kg	Purchased, purity ≥98%	Attenuated impairments in nonspatial memory, reduced brain Aβ plaque deposits, increased neurogenesis in the hippocampus, reduces AD-Related neuronal loss, inflammatory response and microglial activation	Ye et al. (2021)
Pulmonary protection	LPS-induced lung injury	Mice	Sophocarpine, 5 mg/kg	Purchased, purity ≥98%	Reduced lung wet/dry ratio and pathological changes, decreased CD14, IL-6 and TNF-α, increased scavenger receptor class A (SR-A)	Han et al. (2006)
	LPS-induced lung injury	Mice, human lung epithelial A549 cells	Sophocarpine, 12.5, 25, 50 mg/kg, 10, 20, 40 μg/mL	Purchased, purity ≥98%	Reduced lung wet/dry ratio and protein concentration, alleviated LPS-induced lung pathological changes, reduced IL-6, IL-8 production, and NF-κB activation, reduced MDA and MPO, decreased inflammatory cells in broncho-alveolar lavage fluid (BALF), inhibited MAPKs activation and TLR4 expression	Lu et al. (2019)
Renal protection	Isoproterenol (iso)-induced kidney injury	Mice	Sophocarpine, 20, 40 mg/kg	Purchased, purity ≥98%	Reduced kidney injury serum biomarkers SCr, BUN, pathological changes, reduced the release of inflammatory cytokines, inhibited apoptosis, and increased antioxidant protein SOD-1 and SOD-2 expression, decreased fibrotic proteins expression, suppressed TLR-4/NF-kB and TGF-β1/Smad3 signaling pathways, activated Nrf2/HO-1 signaling pathway	Zhou et al. (2022)
	Lupus nephritis	Mice, HEK293 cells	Sophocarpine, 100 mg/kg, 0–800 μg/mL	Purchased	Increase the survival rate and reduced renal injury in MRL/lpr mice, reduced anti-dsDNA antibody and immune complex deposition, suppressed NLRP3 inflammasome formation in MRL/lpr mice, suppressed NF-κB activation	Li et al. (2018)
Morphine withdrawal syndrome	Morphine withdrawal syndrome	Mice	Total extract of *S. alopecuroides* var. *alopecuroides* seeds (100, 200, 300 mg/kg), alkaloid fraction (5, 10, 20 mg/kg)	Extracted and isolated from *S. alopecuroides* L. var. *alopecuroides* seeds	Decreased jumping and diarrhea, the effects of the total extract and alkaloid fraction were not significantly different from methadone	Kianbakht and Hashem Dabaghian (2016)
	Morphine withdrawal syndrome	Human	Alkaloid composition of *S. alopecuroides* extract, 400 mg	Extracted and isolated from *Sophora alopecuroides *L.	Lower COWS score without affecting blood parameters	Kianbakht et al. (2020)
Diabetes	Diabetes	Mice	Sophocarpine, 2.5, 5, 10 mg/kg	Purchased	Attenuated plasma glucose, decreased glycosylated hemoglobin content, triglyceride (TG) and total cholesterol (TC) levels, elevated insulin level, C-peptide level and total Hb content, increased GSH, ceruloplasmin and vitamin E, regulated LDL-c content and HDL-c level and ameliorated pancreatic pathological damage	Su et al. (2021)
Analgesia	Pain	Mice	Sophocarpine,10, 20, 40 mg/kg	Extracted and isolated from *Sophora alopecuroides* L.	Dose-dependent anti-inflammatory effects on carrageenan-induced rat hind paw edema, xylene-induced mouse ear edema and acetic acid-induced mouse vascular permeation	Gao et al. (2009)
	Analgesic and anti-inflammatory effects	Mice	Sophocarpine, 20, 40, 80 mg/kg	Extracted and isolated from *Sophora viciifolia* Hance	Analgesic and anti-inflammatory activities, decreased the expression levels of proinflammatory factors IL-1β, IL-6, and PGE2	Wang et al. (2021b)
	Neuropathic pain	Mice	Sophocarpine, 20, 40 mg/kg	Purchased	Increased mechanical withdrawal threshold (MWT), thermal withdrawal latency (TWL), and tail-curling latency, reduced cold withdrawal threshold (CWT), downregulated HMGB1, TLR4, NF-κB, p-NF-κB, TNF-α, and IL-6 mRNA and protein expression levels in the spinal cord	Jin et al. (2019)

## Formulas containing of sophocarpine

In Chinese medicine, traditional herbal formulas consist of combinations of various herbs that contain multiple active ingredients that work synergistically to exert therapeutic effects. Among these complex formulas, such as the Kaihoujian recipe, Kangfuxiaoyan suppository, and Qingluoyin formula, sophocarpine has been identified as the principal active ingredient.

The Kaihoujian recipe originates from Miao traditional medicine in China and has a long history. It is composed of Shan Dou Gen (*Sophorae Tonkinensis* Radix et Rhizoma—*Sophora tonkinensis* Gagnep. [Fabaceae]), Ba Zhao Jin Long (*Ardisia crispa* [Thunb.] A.DC. [Primulaceae]), Chan Tui (Cicadae Periostracum—*Cryptotympana pustulata* Fabricius [Homoptera]), and Bo He Nao (Menthol—*Mentha haplocalyx* Briq. [Lamiaceae]). Kaihoujian spray, derived from the original formula of Kaihoujian, is primarily employed for the treatment of acute pharyngitis and acute tonsillitis; this spray results in direct mucous membrane absorption, yielding effective outcomes with minimal adverse effects and has gained extensive use. By employing a combination of gray correlation analysis and network pharmacology analysis, Chen et al. further substantiated sophocarpine as the principal active anti-inflammatory constituent in Kaihoujian spray. Kaihoujian spray effectively mitigated NO production in LPS-induced RAW264.7 cells (Chen et al., 2022a). Additionally, Pang’s research group demonstrated that the application of Kaihoujian spray can effectively reduce the count of white blood cells and the levels of inflammatory factors such as IL-1β and MCP-1 in the bloodstream. The therapeutic efficacy of this treatment for acute pharyngitis may be mediated through the PI3K-AKT, NF-κB, and Toll-like receptor signaling pathways (Pang et al., 2023).

Kangfuxiaoyan suppository, a traditional Chinese medicine, is primarily composed of Ku Shen (Radix *Sophorae Flavescentis*—*Sophora flavescens* Aiton [Fabaceae]), Chuan Xin Lian (Andrographis Herba—*Andrographis paniculata* [Burm.f.] Wall. ex Nees [Acanthaceae]), Zi Cao (*Arnebiae Radix*—*Arnebia euchroma* [Royle ex Benth.] I. M. Johnst. and *Arnebia guttata* Bunge [Boraginaceae]), Bai Jiang Cao (Herba Patriniae—*Patrinia scabiosaefolia* Fisch. and *Patrinia villosa* Juss. [Caprifoliaceae]), Pu Gong Ying (*Taraxaci Herba*—*Taraxacum mongolicum* Hand. -Mazz. and *Taraxacum borealisinense* Kitam. [Asteraceae]), Zi Hua Di Ding (*Violae Herba*—*Viola yedoensis* Makino [Violaceae]), Lu Hui (*Aloe Vera*—*Aloe barbadensis* Miller and *Aloe ferox* Miller [Asphodelaceae]), Zhu Dan Fen (Suis Fellis Pulvis—*Sus scrofa domestica* Brisson. [Suidae]). This formulation has been developed for for rectal administration and is marketed in China as a therapeutic option for chronic pelvic inflammatory disease. Zhang et al. conducted metabolomic and network pharmacology analysis to reveal the efficacy of Kangfuxiaoyan suppository in reversing the expression of uterine inflammation markers such as IL-1 and IL-6. Furthermore, it exhibited regulatory effects on pivotal targets such as ARG1, NOS2, NOS3, and its principal constituent sophocarpine, suggesting its potential role in mitigating the inflammatory response (Zhang et al., 2022). Although metabolomics and network pharmacology analysis have shown great potential, this formula needs further exploration in experimental models.

The herbal formula Qingluoyin, composed of Ku Shen (Radix *Sophorae Flavescentis*—*Sophora flavescens* Aiton [Fabaceae]), Qing Feng Teng (Sinomenii Caulis—*Sinomenium acutum* [Thunb.] Rehd et Wils. and *S. acutum* [Thunb.] Rehd et Wils. var. *cinereum* Rehd. et Wils [Menispermaceae]), Huang Bo (Phellodendri Chinensis Cortex—*Phellodendron chinense* C.K. Schneid. [Rutaceae]), Fen Bi Xie (*Dioscoreae Hypoglaucae* Rhizome—*Dioscorea hypoglauca* Palib. [Dioscoreaceae]), is a classic Chinese herbal formula with a clinical application history of more than 40 years, exhibits significant efficacy in treating hot syndrome-related rheumatoid arthritis. The major bioactive compound in Qingluoyin has been identified as sophocarpine, which primarily acts on rheumatoid arthritis by modulating T cells and monocytes through disruption of their interaction. This modulation is achieved by inhibiting the phosphorylation of JNK and p65, resulting in decreased expression of iNOS and IL-1β, thereby suppressing the production of inflammatory factors such as IL-6 and IL-1β (Wang et al., 2021a). Furthermore, Wang discovered that Qingluoyin exerts anti-inflammatory effects in rats with rheumatoid arthritis by upregulating PPAR-γ expression, thereby modulating monocyte/macrophage polarization and adipocyte differentiation (Wang et al., 2021c).

Similarly, the Suduxing formula is also a combination of traditional Chinese herbal medicines, that have been adapted from the potent anti-HIV drug Su-du injection. Liu’s research team discovered that sophocarpine, a major constituent of Suduxing, significantly reduces the levels of HBsAg, HBeAg, and HBV DNA. These findings demonstrated potent anti-HBV activity against both wild-type and entecavir-resistant strains of HBV. Additionally, the observed anti-HBV activity is likely attributed to pivotal molecules including CCNA2, ATF4, FAS, and CDKN1A (Liu et al., 2018).

Compound kushen injection, a traditional Chinese herbal formula, has received approval from the National Medical Products Administration (NMPA) for its efficacy in managing malignant tumor-related pain and bleeding, as well as alleviating chemotherapy-induced adverse reactions. Its mechanism of action involves targeting the transient receptor potential ion channel (TRPV1) pathway. It consists of two medicinal herbs—Ku Shen (Radix *Sophorae Flavescentis*—*Sophora flavescens* Aiton [Fabaceae]), Tu Fu Ling (*Rhizoma Smilacis Glabrae*—*Smilax glabra Roxb.* [Smilacaceae]). The main active ingredients include matrine, oxymatrine, sophocarpine, and oxysophocarpine. Current research increasingly demonstrates the remarkable antitumor effects of compound Kushen injection and its principal constituents, such as matrine, oxymatrine, sophocarpine, and oxysophocarpine (Gao et al., 2021; Yang et al., 2022). Yang et al. performed a clinical meta-analysis and reported that compound Kushen injection effectively relieved liver fibrosis and cirrhosis in hepatitis patients. Then, they used two preclinical animal models and found that Kushen injection (1, 2.5, 5.0, and 7.5 mL/kg) suppressed HSC activation, protecting the liver against hepatic fibrosis and hepatocarcinogenesis by targeting TGF-β/Smad signaling. The authors identified sophocarpine as one of the most potent antifibrotic ingredients in Ku-Shen (Yang et al., 2021b). However, it remains unclear whether other active ingredients, such as oxymatrine, matrine, and oxysophocarpine, synergistically interact with sophocarpine to exert their biological effects. Therefore, further investigations are warranted to elucidate these aspects.

The aforementioned traditional Chinese medicine formulas, which contain sophocarpine as the primary active ingredient, have exhibited diverse therapeutic effects (Table 3). However, further investigations are warranted to ascertain whether sophocarpine has the highest potency among these alkaloids. Moreover, in comparison to individual components, combinations of multiple ingredients may either augment or diminish pharmacological effects. Henceforth, it is imperative to explore the potential interactions between sophocarpine and other compounds along with their specific mechanisms of action in order to elucidate the underlying principles governing traditional Chinese herbal formulae and provide a theoretical foundation for subsequent drug development endeavors.

**TABLE 3:** Traditional Chinese formulas containing sophocarpine

Name	Composition	Main active ingredient	Treating disease	References
Kaihoujian recipe	Shan Dou Gen (*Sophorae Tonkinensis* Radix et Rhizoma—*Sophora tonkinensis* Gagnep. [Fabaceae]), Ba Zhao Jin Long (*Ardisia crispa* [Thunb.] A.DC. [Primulaceae]), Chan Tui (Cicadae Periostracum—*Cryptotympana pustulata* Fabricius [Homoptera]), Bo He Nao (Menthol—*Mentha haplocalyx* Briq. [Lamiaceae])	Bergeninum, matrine, sophocarpine, trifolirhizin, genistein	Acute pharyngitis and acute tonsillitis	(Chen et al., 2022a; Pang et al., 2023)
Kangfuxiaoyan suppository	Ku Shen (Radix *Sophorae Flavescentis*—*Sophora flavescens* Aiton [Fabaceae]), Chuan Xin Lian (Andrographis Herba—*Andrographis paniculata* [Burm.f.] Wall. ex Nees [Acanthaceae]), Zi Cao (*Arnebiae Radix*—*Arnebia euchroma* [Royle ex Benth.] I. M. Johnst. and *Arnebia guttata* Bunge [Boraginaceae]), Bai Jiang Cao (Herba Patriniae—*Patrinia scabiosaefolia* Fisch. and *Patrinia villosa* Juss. [Caprifoliaceae]), Pu Gong Ying (*Taraxaci Herba*—*Taraxacum mongolicum* Hand. -Mazz. and *Taraxacum borealisinense* Kitam. [Asteraceae]), Zi Hua Di Ding (*Violae Herba*—*Viola yedoensis* Makino [Violaceae]), Lu Hui (*Aloe Vera*—*Aloe barbadensis* Miller and *Aloe ferox* Miller [Asphodelaceae]), Zhu Dan Fen (Suis Fellis Pulvis—*Sus scrofa domestica* Brisson. [Suidae])	Matrine, sophocarpine, aloin, esculetin-O-glucuronide, 7,4′-dihydroxyisoflavone-O-glucuronide, 4′-methoxyisoflavone-7-O-glucuronide	Chronic pelvic inflammatory disease	Zhang et al. (2022)
Qingluoyin formula	Ku Shen (Radix *Sophorae Flavescentis*—*Sophora flavescens* Aiton [Fabaceae]), Qing Feng Teng (Sinomenii Caulis—*Sinomenium acutum* [Thunb.] Rehd et Wils. and *Sinomenium acutum* [Thunb.] Rehd et Wils. var. *cinereum* Rehd. et Wils [Menispermaceae]), Huang Bo (Phellodendri Chinensis Cortex—*Phellodendron chinense* C.K. Schneid. [Rutaceae]), Fen Bi Xie (*Dioscoreae Hypoglaucae* Rhizome—*Dioscorea hypoglauca* Palib. [Dioscoreaceae])	Matrine, sinomenine, sophocarpine, palmatine, berberine, diosgenin	Rheumatoid arthritis	(Wang et al., 2021a; Wang et al., 2021c)
Suduxing	--	Matrine, oxymatrine, chlorogenic acid, sophocarpine, baicalein, wogonin	HBV infection	Liu et al. (2018)
Compound kushen injection	Ku Shen (Radix *Sophorae Flavescentis*—*Sophora flavescens* Aiton [Fabaceae]), Tu Fu Ling (*Rhizoma Smilacis Glabrae*—*Smilax glabra Roxb.* [Smilacaceae])	Matrine, oxymatrine, sophocarpine, oxysophocarpine, macrozamin, sophoridine, piscidic acid, trifolirhizin	Chemotherapy-induced adverse reactions, cancer	(Gao et al., 2021; Yang et al., 2022)

## Association of sophocarpine with inflammatory cytokines, NF-κB signaling, and p38MAPK signaling

### Sophocarpine regulates inflammatory cytokines

Inflammation is an immune response triggered by various harmful stimuli. A properly regulated inflammatory response can effectively shield the body against external stimuli, whereas an excessive inflammatory response can exacerbate the initial injury and consequently mediate a range of pathophysiological processes (Dinarello, 2010). Cytokines serve as crucial immune mediators that regulate inflammatory responses in diverse diseases and are primarily categorized into proinflammatory cytokines and anti-inflammatory cytokines. Among these cytokines, common proinflammatory cytokines, such as TNF-α, IL-6, and IL-1β, predominantly contribute to the progression of inflammation in diseases; conversely, anti-inflammatory cytokines, such as IL-10, IL-4, and transforming growth factor beta (TGF-β), exert opposing effects (Yahfoufi et al., 2018). The dynamic equilibrium between proinflammatory cytokines and anti-inflammatory cytokines constitutes a vital component for maintaining immune homeostasis within the body (Khatami et al., 2016). Sophocarpine has been demonstrated to modulate various proinflammatory and anti-inflammatory cytokines through multiple pathways, exerting significant biological effects. In the context of inflammatory diseases affecting the osteoarticular system (Zhu and Zhu, 2017; Wu et al., 2019) and colitis models (Wang et al., 2012; Zhang et al., 2015), sophocarpine primarily exerts its anti-inflammatory effects by downregulating the levels of key proinflammatory factors such as IL-6, TNF-α, IL-1β, and IL-12. However, in a mouse model of asthma investigated by Zhi et al., sophocarpine was found to mitigate lung injury by modulating the expression of IL-4, IL-5, and INF-γ (Zhi et al., 2021). Furthermore, in LPS-induced lung injury models, sophocarpine predominantly targets the regulation of IL-6, TNF-α, and IL-8 as major therapeutic targets (Han et al., 2006; Lu et al., 2019), demonstrating its multifaceted therapeutic potential. In addition, Sang et al. (Sang et al., 2017) demonstrated that sophocarpine exhibited antiviral and hepatoprotective effects in a Concanavalin A-induced hepatitis mouse model by reducing the levels of the proinflammatory cytokines IFN-γ and TNF-α. In nonviral liver diseases such as septic liver injury (Zhengyu et al., 2018; Hou et al., 2020) and nonalcoholic steatohepatitis (Song et al., 2011; Song et al., 2013), sophocarpine exerts its liver protective effects through the modulation of key inflammatory cytokines, including IL-6, TNF-α, TGF-β1, and IL-1β. In the field of cardiovascular disease, sophocarpine effectively modulates proinflammatory mediators, including TGF-β, IL-6, IL-1β, and TNF-α, and proinflammatory vascular adhesion molecules, such as VCAM-1 and ICAM-1. This regulation contributes to the therapeutic potential of sophocarpine in treating atherosclerosis (Fang et al., 2020) and restenosis after angioplasty (Yang et al., 2020; Yang et al., 2021a). Few studies have explored the impact of sophocarpine on tumors through its modulation of inflammatory cytokines. Nevertheless, evidence suggests that sophocarpine can mitigate cancer-induced cachexia by concurrently reducing the levels of TNF-α and IL-6 in RAW264.7 cells and macrophages (Zhang et al., 2008), indicating the possible involvement of inflammatory cytokines in the antitumor effects exerted by sophocarpine. Further investigations are warranted to elucidate the precise underlying mechanisms involved.

### Sophocarpine and NF-κB signaling

Nuclear factor kappa B (NF-κB) is a transcription factor family that orchestrates inflammatory responses and governs diverse functions, including immunity, cell proliferation, and cell differentiation (Mitchell et al., 2016). The NF-κB pathway is involved in numerous diseases characterized by inflammation, including cancer and ischemia‒reperfusion injury in various organs (Guijarro and Egido, 2001; Hoesel and Schmid, 2013). Concurrently, an increasing body of research has demonstrated that herbal ingredients can elicit multiple biological effects, such as anti-inflammatory effects, antitumor activity, and organ protection, through modulation of the NF-κB signaling cascade (Ye et al., 2020; Dong et al., 2022; Lin et al., 2022). Currently, sophocarpine has emerged as a potent modulator that targets this pivotal NF-κB pathway to exert its therapeutic effects across diverse disease contexts. In the context of the nervous system, sophocarpine has been shown to enhance pain tolerance toward various stimuli in a mouse model by downregulating NF-κB phosphorylation levels, thereby exhibiting therapeutic potential for neuropathic pain (Jin et al., 2019). In terms of specific nerve cells, Zhu et al. demonstrated that sophocarpine intervention in a β-amyloid injury model established in PC12 cells can mitigate damage caused by β-amyloid through NF-κB nuclear translocation, further substantiating the association between sophocarpine and neuroprotection via the NF-κB pathway (Zhu et al., 2021). In addition to its neuroprotective effects, sophocarpine has been shown to have protective effects against septic liver injury (Zhengyu et al., 2018), nonalcoholic steatohepatitis (Song et al., 2015), and LPS-induced lung injury (Lu et al., 2019) through the inhibition of NF-κB and JNK-related pathways. This mechanism leads to a reduction in inflammatory factors within the body, alleviation of oxidative stress, and ultimately safeguards the liver and lungs from damage caused by relevant detrimental factors. In the context of kidney function, NF-κB serves as a pivotal pathway for sophocarpine in addressing isoproterenol (iso)-induced kidney injury (Zhou et al., 2022) while also exhibiting potential in managing autoimmune disorders such as lupus nephritis. By diminishing the production of anti-dsDNA antibodies and reducing immune complex deposition within renal tissues, NF-κB significantly contributes to the therapeutic efficacy of sophocarpine treatment (Li et al., 2018). Moreover, in the context of heart disease, sophocarpine not only ameliorates ischemia‒reperfusion injury resulting from postinterventional treatment for myocardial infarction (Li et al., 2011) but also exhibits a favorable mitigating effect on restenosis following revascularization. The therapeutic application of sophocarpine in these two conditions involves modulation of the NF-κB pathway and ultimately aims to reduce inflammatory mediators such as IL-6 and IL-1β (Yang et al., 2021a). In conclusion, NF-κB serves as a pivotal signaling molecule through which sophocarpine exerts multiorgan-protective and anti-inflammatory effects and combats autoimmune diseases. Additional investigations into the interplay between sophocarpine and NF-κB in alternative pathological states are warranted.

### Sophocarpine and p38MAPK signaling

The mitogen-activated protein kinase (MAPK) signaling pathway plays a crucial role in regulating diverse biological processes within the human body, including cell survival, differentiation, proliferation, inflammation, and apoptosis (Pearson et al., 2001). Among the MAPKs identified in organisms, three prominent ones include signal-regulated kinase (ERK), c-Jun N-terminal kinase (JNK), and p38 kinase (Wei et al., 2021). Specifically, the p38MAPK signaling pathway, which is an integral member of the MAPK family consisting of four subtypes (P38α, P38β, P38γ, and P38δ), actively participates in essential mechanisms such as the regulation of inflammation, the induction of cellular apoptosis, and the modulation of autophagy (Han et al., 1994). Moreover, the p38MAPK signaling pathway is highly important for exploring the potential of traditional Chinese medicine extracts, and an increasing body of research has revealed promising associations between traditional Chinese medicine and this pathway (Chang and Xiong, 2020; Li et al., 2023), including sophocarpine. In a murine model of septic liver injury, sophocarpine effectively suppressed the p38/JNK signaling pathway, mitigated oxidative stress, and upregulated the expression of anti-inflammatory factors such as superoxide dismutase (SOD), catalase (CAT), and glutathione (GSH) to exert hepatoprotective effects (Zhengyu et al., 2018). In addition, sophocarpine is closely associated with the p38MAPK signaling pathway in cardiovascular diseases. Li et al. conducted a study on rats to investigate the effects of sophocarpine on myocardial ischemia‒reperfusion and reported a reduction in the phosphorylation of P38/JNK and decreased infiltration of central granulocytes, myeloperoxidase (MPO) activity, and infarct area (Li et al., 2011). Similarly, Liu et al. evaluated the impact of sophocarpine on endothelial apoptosis by isolating rat aortas and culturing rat aortic endothelial cells. They discovered that sophocarpine exerts antiapoptotic effects through p38MAPK/Nrf2 signaling by reducing ROS production, which is potentially regulated upstream by MKK3/6 (Liu et al., 2017b). Yang et al. obtained similar results in their intervention with sophocarpine in a rat restenosis after angioplasty model and observed a decrease in IL-1β and TNF-a levels involved in its vascular protective effect (Yang et al., 2020). Additionally, in their investigation of the therapeutic potential of sophocarpine against tumors, Liu et al. discovered that sophocarpine effectively hinders the maturation of miRNA-21 through the p38MAPK signaling pathway, thereby suppressing the proliferation, invasion, and migration of head and neck squamous cell carcinoma (HNSCC) (Liu et al., 2017a). Based on these findings, it can be inferred that while the precise relationship between sophocarpine and the p38MAPK signaling pathway remains unclear in other disease contexts, it is evident that this pathway plays a crucial role in mediating the biological effects of sophocarpine.

### Pharmacokinetics

Pharmacokinetic research is crucial for the application and prospects of sophocarpine. After a single oral administration of 200 mg/kg of sophocarpine to fasting rabbits, the C_max_ was 11.64 ± 1.28 mg L^−1^, the T_max_ was 40.95 ± 8.35 min, and the AUC was 1,475.72 ± 326.5 mg·min/L, respectively (Bao-xin, 2009). While in another study, rapid intravenous injection of 10 mg/kg sophocarpine in rabbits resulted in rapid distribution in the body, with a half-life of 6.0 min, an elimination half-life of 82.6 min, and a pseudoequilibrium time of approximately 30 min. The mean residence time (MRT) was 106 min, and the mean volume of distribution was 2.992 L/kg. The drug was found to be distributed specifically, primarily in the peripheral compartment (Liu et al., 1987). When administered via tail vein injection to rats, sophocarpine exhibited widespread distribution in the body, with peak tissue concentrations observed within 5–20 min, and the highest levels found in the kidneys, followed by the liver, gastrointestinal tract, and lungs. Detectable levels in the brain and adipose tissues suggested penetration of the blood‒brain barrier (Liu and Huang, 1988). The plasma clearance rate was 9.15 ± 2.58 mL/min, with 32.0% ± 7.0% of the excreted sophocarpine remaining unchanged in the urine within 24 h, indicating a renal clearance fraction of 0.32 ± 0.07. The hepatic and renal clearance rates were 6.21 and 2.94 mL/min, respectively (Liu and Huang, 1988). Sophocarpine was found to inhibit the activity of CYP3A4 and 2C9 (Lin et al., 2015; Zhang et al., 2019), leading to significant alterations in the pharmacokinetic parameters of coadministered drugs such as umbralisib. However, Weng et al. reported that co-administration of umbralisib with sophocarpine led to a significant increase in the metabolism of umbralisib in rats, resulting in alterations to the pharmacokinetic parameters of umbralisib. Specifically, there were significant reductions in the area under the curve for plasma concentration from zero to last measurable plasma sample time (AUC_0-t_) and the area under the curve for plasma concentration from zero to infinity (AUC_0-∞_), as well as significant decreases in C_max_ and T_max_ (Weng et al., 2022b). These alterations cannot be attributed to inhibition of CYP3A4 and 2C9 activity, indicating a potential interaction between sophocarpine and umbralisib that warrants further investigation in future studies.

To improve the absorption and bioavailability of sophocarpine, one study utilized an exosome delivery system containing sophocarpine from *Sophora* extract, which enables enhanced penetration through the stratum corneum into deeper layers of the skin, resulting in significantly greater drug flux and absorption rate (Zhou et al., 2010b). Peng et al. discovered that the traditional Chinese medicinal formula, Kushen recipe, containing sophocarpine, exhibited limited permeability in aqueous solution but showed potential for treating skin-related conditions such as pruritus and eczema. They conducted a study to assess the transdermal parameters of the four main alkaloids (matrine, oxymatrine, sophocarpine and oxysophocarpine) in Kushen recipe. They observed that oxymatrine and oxysophocarpine exhibited strong polarity and poor lipid solubility, leading to low skin permeability. To address this issue, the authors employed techniques involving ethanol extraction and purification with absorbent resin to obtain purer alkaloids from *Sophora flavescens* Ait. These purified alkaloids were then combined with carbomer and essential oil to formulate a gel agent. The essential oil used was extracted from *Schizonepeta tenuifolia* Briq, containing main components such as menthone, menthol, and pulegone which enhanced the percutaneous efficacy of *Sophora* alkaloids. Ultimately, these methods resulted in improved permeability of Kushen recipe and significantly enhanced transdermal parameters of the four alkaloids in rat experiments after 48 h (Peng et al., 2007). Furthermore, a patented cocrystal compound has jointly improved the water solubility of rhein and sophocarpine, thereby enabling better absorption and more rapid attainment of effective drug blood concentration for enhanced therapeutic effects of the medication (Lv et al., 2024). While specific pharmacokinetic parameters were not explicitly mentioned in some articles, considering the various forms of entry of sophocarpine into the body, we have summarized the information about related parameters in the table below (Table 4).

**TABLE 4:** Pharmacokinetic parameters of sophocarpine

Forms/Dose	Administration	Mentioned parameters	Animal	References
Sophocarpine, 200 mg/kg	Intragastric	C_max_ = 11.64 ± 1.28 mg·L^−1^; T_max_ = 40.95 ± 8.35 min; AUC_0-t_ = 1,475.72 ± 326.5 mg·min/L; t_1/2α_ = 28.92 min; t_1/2β_ = 90.18 min	Rabbit	Bao-xin (2009)
Sophocarpine, 10 mg/kg	Intravenous	V_c_ (volume of the central compartment) = 1.40 L/kg; CL (clearance) = 28.2 mL/(kg·min^−1^); MRT = 106 min; t_1/2α_ = 6.0 min; t_1/2β_ = 82.6 min	Rabbit	Liu et al. (1987)
Sophocarpine, 110 μg/min	Intravenous	CL = 9.15 ± 2.58 mL/min	Rat	Liu and Huang (1988)
Water extract of *Sophora flavescens* Ait., 2 mg/mL	Transdermal	Q_48h_ (48-h cumulative transdermal dose) = 47.3709 ± 9.4619 μg/cm^2^; Transdermal rate = 1.02 μg·cm^2^/h	Rat	Peng et al. (2007)
Purified product of *Sophora flavescens* Ait., 2 mg/mL	Transdermal	Q_48 h_ = 102.7327 ± 22.7135 μg/cm^2^; Transdermal rate = 2.16 μg·cm^2^/h	Rat	Peng et al. (2007)
Kushen recipe gel, 1 g	Transdermal	Q_48h_ = 395.6320 ± 169.4830 μg/cm^2^; Transdermal rate = 8.41μg·cm^2^/h	Rat	Peng et al. (2007)
Yanshu administration, 1.2 g/kg	Intravenous	AUC_0-t_ = 378.30 ± 107.07 μg·h/L; AUC_0-∞_ = 420.86 ± 123.99 μg·h/L; C_max_ = 721.52 ± 168.60 μg/L; T_max_ = 0. 04 ± 0.02 h	Beagle dog	Liu et al. (2012)
(*E*)-12-*N*-(*m*-cyanobenzenesulfonyl)-β,γ-sophocarpinic acid, 25 mg/kg	Intragastric	T_max_ = 0.5 ± 0.00 h; C_max_ = 4.54 ± 0.76 μM; AUC_0–24 h_ = 7.29 ± 0.71 μM·h; MRT = 1.53 ± 0.14 h; t_1/2_ = 1.17 ± 0.02 h	Rat, mice	Gao et al. (2013)

Although sophocarpine can be absorbed in various forms to exert its pharmacological effects, current pharmacokinetic studies are predominantly conducted via intravenous administration, and the pharmacokinetic parameters for oral administration are still lacking and warrant further exploration. Additionally, like other drugs, sophocarpine requires further investigation to improve its *in vivo* efficacy; for instance, sophocarpine can be absorbed through the skin, thus exerting antipruritic and anti-inflammatory effects, and future efforts should aim to enhance its subcutaneous absorption, which is currently low. Furthermore, the insufficiency of clinical trials on sophocarpine in mammals and humans needs to be addressed to facilitate its clinical application.

#### Toxicity

**FIGURE 4:**
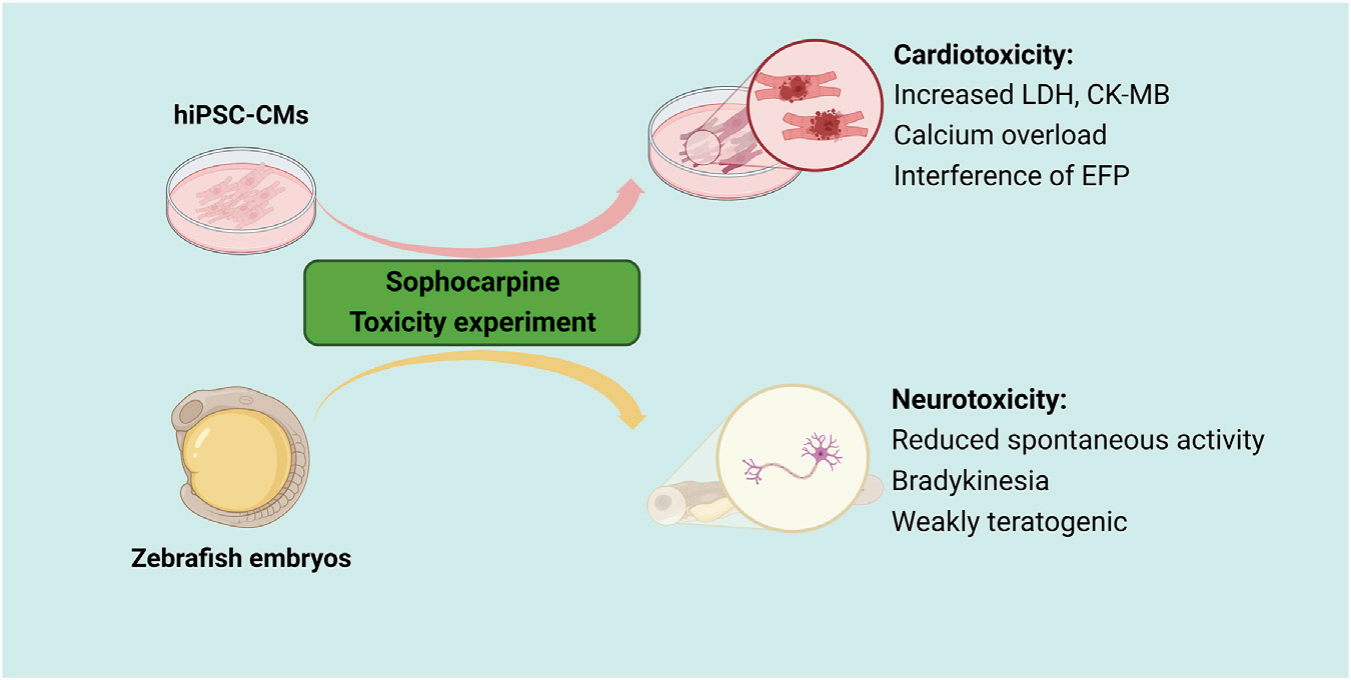
Neurotoxicity and cardiotoxicity of sophocarpine. When zebrafish embryos are exposed to sophocarpine, their behavioral assessments show a decrease in activity and coordination, along with abnormal movement and swimming performance. In cardiac muscle cells, the cardiotoxicity of sophocarpine is associated with disruptions in calcium homeostasis and oxidative stress, leading to pathological apoptosis. These findings collectively indicate that sophocarpine may have adverse effects on the nervous system and the heart.

Sophocarpine is a major ingredient in many traditional Chinese medicines. It has been widely used in China. However, the toxicity and safety profile of sophocarpine have not been well characterized to date. In general, it has been found that sophocarpine potentially induces neurotoxicity and cardiotoxicity (Figure 4). Lu et al. studied the developmental toxicity and neurotoxicity of sophocarpine in zebrafish embryos/larvae and reported that sophocarpine had neurotoxic effects with ED_50_ and LD_50_ values of 87.1 and 166 mg/L, respectively. It induces teratogenic and lethal effects on zebrafish embryos, alters spontaneous movement and inhibites swimming behavior (Lu et al., 2014). Moreover, using a cardio nonlabeled cell function analysis and culture system (Cardio-NLCS), Wang et al. showed that sophocarpine dose-dependently affects the impedance and extracellular field potential (EFP) of human-induced pluripotent stem cell-derived cardiomyocytes (hiPSC-CMs), suggesting that sophocarpine may have cardiotoxic effects. Furthermore, the mechanism was associated with the disturbance of calcium homeostasis and oxidative stress (Wang et al., 2019b).

In the future, toxicological research on sophocarpine must continue to occur to ensure its safety and efficacy in clinical applications. Although initial experiments have demonstrated its therapeutic potential, the chronic toxicity associated with long-term use, particularly its potential impact on the liver and kidneys, remains unclear. It will be necessary to further explore the dose‒response relationship of sophocarpine, and metabolic changes following prolonged exposure, and how this relationship affects specific biomarkers and molecular pathways.

## Discussion and future prospects

Sophocarpine can be found in various types of traditional herbal medicines. It is a well-recognized matrine-type quinolizidine alkaloid with an established chemical structure. A large number of studies have confirmed its potential pharmacological benefits. This review systematically summarized the pharmacological activities of sophocarpine. Sophocarpine has beneficial effects on various cancer types, such as lung cancer, gastric cancer, colon cancer, cervical cancer, prostate cancer, liver cancer, myeloma, and head and neck cancer. The mechanism may involve signaling pathway inactivation or apoptosis inhibition. In terms of inflammation, sophocarpine has strong anti-inflammatory effects on pharyngitis, rheumatoid arthritis, osteoarthritis, colitis and toxicity-induced inflammatory responses. Sophocarpine has excellent antiviral effects on viral hepatitis, coxsackievirus, and enterovirus 71. Although sophocarpine has significant pharmacological effects, its clinical value has not been fully elucidated. Most of the related reports were observational rodent or cell culture studies, and clinical explorations of the pharmacological activities of these materials are not sufficient. Moreover, pharmacological parameters, such as dose range, minimum active concentration and duration still merit further exploration. Therefore, to promote the clinical application of sophocarpine, different human disease models or various clinical occasions may be needed to improve the clinical efficacy of sophocarpine therapy. Additionally, although sophocarpine has been proven to exert protective effects on multiple organs, it is still unclear whether it can be effective for treating other specific organ diseases. In the future, this protective effect will need to be further validated in various disease models. In addition to *in vitro* and *ex vivo* studies, positive or negative control experiments and experiments on large animals are needed due to differences between humans and rodents.

In terms of pharmacokinetics, it has been found in an article that the distribution of sophocarpine within the body of rabbits conforms to a two-compartment model (Liu et al., 1987). It has a short half-life and is primarily excreted through urine. After intravenous injection, it is widely distributed in various tissues within the body, and thereby has protective effects on multiple organs. However, the high accumulation of NGAL in renal tissues suggests the need for cautious consideration when administering this compound to patients with renal impairment (Liu and Huang, 1988). Currently, there is limited research on the metabolism and transformation of sophocarpine within the body, and further study is necessary to determine its pharmacokinetic characteristics and design different forms of biological preparations to enhance the activity and protective properties of sophocarpine. Additionally, studies have revealed that sophocarpine can alter the pharmacokinetic properties of the antitumor drug umbralisib in mice (Weng et al., 2022b), indicating the need for future research on herb-drug interactions involving sophocarpine to improve the concentration of corresponding drugs in the body or to prevent excessive accumulation.

Furthermore, the reported pharmacological studies regarding organ protection by sophocarpine have focused mainly on cardiovascular diseases. Scattered studies have indicated that sophocarpine may protect other vital organs, such as the liver, kidney, brain, and lung. There are different types of organ injury, such as ischemia/reperfusion injury, toxicity-induced acute organ injury, and transfusion-induced organ injury. Current studies have been conducted only on several specific types of injuries, and there are still large knowledge gaps that need to be filled in future studies.

A clinically feasible agent must have been tested in both clinical and toxicological studies. The promising safety and toxicity of the candidate agent have yet to be assessed. However, there are a limited number of studies on sophocarpine-induced toxicity in the current review. One study focused on developmental toxicity and neurotoxicity in zebrafish (*Danio rerio*) embryos/larvae (Lu et al., 2014), while another researcher studied cardiotoxic effects in human-induced pluripotent stem cell-derived cardiomyocytes (Wang et al., 2019b). Notably, other alkaloids, such as matrine and sophoridine, were reported to be hepatotoxic, to cause developmental toxicity, neurotoxicity and reproductive toxicity (Li et al., 2021; Wang et al., 2022b). Therefore, because of their toxicity, the therapeutic application of these alkaloids must be taken seriously. Unfortunately, it is currently unknown whether sophocarpine has toxic effects *in vivo*. In addition, there is no information regarding target organ toxicity across the species. Moreover, it would be interesting to determine whether combination medication would offer a more potent therapeutic effect while reducing toxicity and complications.

Although the pharmacological effect of sophocarpine was described in this review, current preclinical works have focused mainly on verifying the therapeutic effect of sophocarpine on various disease models or cell lines. Most of those studies were performed without sufficient mechanistic studies. Identification of the target molecular mechanism responsible for the pharmacological action will aid in the development of new therapeutic strategies. However, the target or mechanism of action is not clearly defined in most related studies. The lack of mechanistic insight, however, seems to be a common issue for sophocarpine-based studies. We believe that a better understanding of the mechanism underlying the pharmacological response is vital for promoting the clinical application of sophocarpine.
